# Gene Essentiality Analyzed by *In Vivo* Transposon Mutagenesis and Machine Learning in a Stable Haploid Isolate of *Candida albicans*

**DOI:** 10.1128/mBio.02048-18

**Published:** 2018-10-30

**Authors:** Ella Shtifman Segal, Vladimir Gritsenko, Anton Levitan, Bhawna Yadav, Naama Dror, Jacob L. Steenwyk, Yael Silberberg, Kevin Mielich, Antonis Rokas, Neil A. R. Gow, Reinhard Kunze, Roded Sharan, Judith Berman

**Affiliations:** a School of Molecular Cell Biology and Biotechnology, Department of Molecular Microbiology and Biotechnology, George Wise Faculty of Life Sciences, Tel Aviv University, Ramat Aviv, Israel; b School of Medical Sciences, Institute of Medical Sciences, University of Aberdeen, Aberdeen, United Kingdom; c Department of Biological Sciences, Vanderbilt University, Nashville, Tennessee, USA; d Institute of Biology, Dahlem Centre of Plant Sciences, Freie Universität Berlin, Berlin, Germany; e The Blavatnik School of Computer Science, Raymond & Beverly Sackler Faculty of Exact Sciences, Tel Aviv University, Tel Aviv, Israel; Universidad de Córdoba; University of Minnesota; Friedrich Schiller University Jena

**Keywords:** *Candida albicans*, genome analysis, genomics, machine learning, phenotypic identification, transposons

## Abstract

Knowing the full set of essential genes for a given organism provides important information about ways to promote, and to limit, its growth and survival. For many non-model organisms, the lack of a stable haploid state and low transformation efficiencies impede the use of conventional approaches to generate a genome-wide comprehensive set of mutant strains and the identification of the genes essential for growth. Here we report on the isolation and utilization of a highly stable haploid derivative of the human pathogenic fungus Candida albicans, together with a modified heterologous transposon and machine learning (ML) analysis method, to predict the degree to which all of the open reading frames are required for growth under standard laboratory conditions. We identified 1,610 C. albicans essential genes, including 1,195 with high “essentiality confidence” scores, thereby increasing the number of essential genes (currently 66 in the Candida Genome Database) by >20-fold and providing an unbiased approach to determine the degree of confidence in the determination of essentiality. Among the genes essential in C. albicans were 602 genes also essential in the model budding and fission yeasts analyzed by both deletion and transposon mutagenesis. We also identified essential genes conserved among the four major human pathogens C. albicans, Aspergillus fumigatus, Cryptococcus neoformans, and Histoplasma capsulatum and highlight those that lack homologs in humans and that thus could serve as potential targets for the design of antifungal therapies.

## Abstract (precis)

Comprehensive understanding of an organism requires that we understand the contributions of most, if not all, of its genes. Classical genetic approaches to this issue have involved systematic deletion of each gene in the genome, with comprehensive sets of mutants available only for very-well-studied model organisms. We took a different approach, harnessing the power of *in vivo* transposition coupled with deep sequencing to identify >500,000 different mutations, one per cell, in the prevalent human fungal pathogen Candida albicans and to map their positions across the genome. The transposition approach is efficient and less labor-intensive than classic approaches. Here, we describe the production and analysis (aided by machine learning) of a large collection of mutants and the comprehensive identification of 1,610 C. albicans genes that are essential for growth under standard laboratory conditions. Among these C. albicans essential genes, we identify those that are also essential in two distantly related model yeasts as well as those that are conserved in all four major human fungal pathogens and that are not conserved in the human genome. This list of genes with functions important for the survival of the pathogen provides a good starting point for the development of new antifungal drugs, which are greatly needed because of the emergence of fungal pathogens with elevated resistance and/or tolerance of the currently limited set of available antifungal drugs.

## INTRODUCTION

The complete set of genes that are essential for survival and growth of eukaryotes are currently known for only a few model eukaryotes, such as the budding yeast Saccharomyces cerevisiae and the fission yeast Schizosaccharomyces pombe (1–4). Gene essentiality is of critical interest in the case of pathogenic fungi; the set of essential genes that are conserved across pathogens, and not in their hosts, are candidate targets for broad-spectrum antifungal drugs. Genes specific for a smaller group of pathogens are candidate targets for more-specific applications. The identification of essential genes as antifungal targets for new classes of antifungals is critical because of the rapid emergence and spread of resistant or tolerant isolates and species in organisms treated with the currently available antifungal drugs (5–7).

Many human fungal pathogens lack a complete sexual cycle, making it difficult to perform classical genetic crosses that validate gene segregation. A classic example is Candida albicans, a member of the normal human microbiome and the most common cause of human fungal nosocomial infection (8). C. albicans generally grows as a heterozygous diploid organism. We recently identified C. albicans haploids, which arise via mitotic chromosome loss events rather than meiosis, providing a critical tool for the genetic analysis of this important pathogen (9).

In haploid model organisms, classic studies test gene essentiality by the analysis of meiotic segregants (2); linkage of a marker to the inability to grow as a haploid provides definitive proof of gene essentiality (10). Such approaches are not applicable to many pathogenic fungi, especially those that do not undergo conventional meiosis.

Much effort has been invested in constructing libraries of C. albicans mutant isolates via the use of directed deletions (11–14), induced deletions (15), or *in vitro* transposon (Tn) insertions (16–18) and by repression of expression from a single, regulatable copy of the gene of interest (19–21). In addition, the UAU1 system, which couples *in vitro* transposition with a double-selection scheme to select for homozygosis of the insertion allele (22), identified several hundred genes listed as “likely essential” or “possibly essential,” on the basis of failure to detect homozygosis (203 genes). Clustered regularly interspaced short palindromic repeat (CRISPR)/Cas9 drive systems make the gene deletion/disruption process more efficient (23–26). Yet all of those approaches rely upon transformation to generate each individual mutant, which often becomes the bottleneck for generating complete sets of mutant libraries. Despite all of these efforts, only 66 C. albicans genes are currently listed as “essential,” “essential for viability,” “essential for growth,” “essential protein,” or “plays an essential role during mitotic growth” under standard growth conditions in the Candida Genome Database (CGD) (27). However, such tests of essentiality are sensitive to growth conditions and the methods used to assess growth, leading to ambiguity in the literature regarding which C. albicans genes are essential for viability under laboratory conditions.

An alternative approach is to determine gene essentiality using *in vivo* transposition. In prokaryotes, transposon sequencing (TnSeq) involves the transformation of a transposon-transposase complex, which can generate millions of mutants in a single transformation, coupled with high-throughput sequencing that analyzes all of the transposon insertion sites (28–30). In a recent example, Tnseq phenotypic analysis of 32 bacterial species assigned >2,000 poorly annotated genes to specific functional groups (31). Importantly, while this approach is extremely efficient and valuable for genotype/phenotype analyses in prokaryotes, it cannot be used in eukaryotes.

In the model yeast S. cerevisiae, a heterologous maize *Activator*/*Dissociation* (*Ac*/*Ds*) element (32) was induced to transpose (33) and was harnessed to rapidly generate a large scale SATAY (SAturated Transposon Analysis in Yeast) library of insertion mutants (34). In the fission yeast S. pombe, genome saturating insertion mutagenesis performed with the Hermes transposon yielded >350,000 independent insertions (35). In the filamentous pathogenic fungus Aspergillus fumigatus, a smaller-scale analysis performed with the *Impala* transposon (from Fusarium oxysporum) identified 96 essential genes (36). These approaches identify recessive mutations in haploid organisms.

Here we generated a stable haploid C. albicans isolate carrying an *Ac* transposase/*Ds-NAT1* two-element system (33) to implement an *in vivo* transposition approach for studying this important pathogen. We used the system to identify genes important for growth under standard laboratory conditions and developed a machine learning (ML) approach to infer essentiality/nonessentiality, i.e., the ability to grow under standard laboratory conditions, in an unbiased fashion. We also applied the ML approach to data from S. cerevisiae and S. pombe transposon studies and then utilized the results to identify a core set of orthologs essential in all three yeasts in deletion and transposon studies. We provide a comprehensive, genome-wide assessment of gene essentiality, a confidence measure of gene essentiality/nonessentiality on the basis of the range of studies that have been performed with each gene, and we highlight essential genes that are not conserved in humans and that can serve as potential targets for the development of new antifungal drugs.

## RESULTS

### Selection of a stable haploid strain carrying the *Ac*/*Ds* system.

We constructed transposon insertion libraries in a haploid C. albicans isolate (YJB-T900) by sequential insertion of a codon-optimized *Ac* transposase (*Ac*TPase4xCa) at the neutral *NEUT5L* locus and a *Ds-NAT1* transposon such that it interrupted the *ADE2* promoter region (37). After insertion of the *Ac* transposase, single colonies were checked for DNA content by flow cytometry. Among the colonies, 50% (6/12) were haploid and the others consisted of mixed populations of haploid and diploid cells. One all-haploid colony (YJB-T1792) was then transformed with *Ds-NAT1*. The DNA content was retested, and 58% (14/24) of the colonies were found to be haploid; one of them (YJB-T1081) was archived, restreaked, and rearchived (YJB-T1082). Like the original haploids, these haploids showed reduced virulence relative to strain SC5314, the heterozygous laboratory strain from which they were derived (see Fig. S1A in the supplemental material).

10.1128/mBio.02048-18.2 FIG S1 Virulence of haploid reads, transposon insertion read maps, and machine learning performance. Download FIG S1, TIF file, 0.2 MB. Copyright © 2018 Shtifman Segal et al. 2018 Shtifman Segal et al. This content is distributed under the terms of the Creative Commons Attribution 4.0 International license.

**Fig 1:**
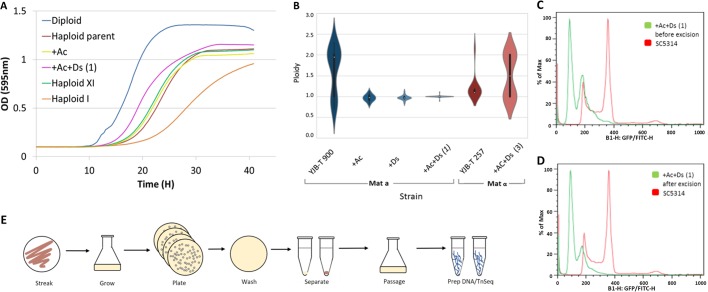
Strain and Tn library construction. (A) Growth curves for strains are indicated as follows: blue, diploid (SC5314); red, haploid *MAT***a** parent (YJB-T900); yellow, +Ac (YJB-T 1792); pink, +Ac+Ds(1) (YJB-T1081) (growth of YJB-T1082 [also +Ac+Ds] was indistinguishable from that of YJB-T1081); orange, haploid I (9); green, haploid XI (9). All growth curve analyses were performed in triplicate, with standard deviations ranging from 0 to 0.05. OD, optical density. (B) Summary of DNA content for multiple isolates for each strain (*n* = 24) [+Ds (YJB-T1794); *MATα* haploid parent YJB-T257; +Ac+Ds(3); YJB-T2743]. (C and D) Flow cytometric DNA content for strain YJB-T1081 before (C) and after (D) excision of *Ds-NAT1*. (E) Schematic of transposon mutant library preparation process.

Importantly, retesting of hundreds of single colonies from YJB-T1081 and YJB-T1082 from the archived stock always produced stable haploids, in contrast to its semistable parent (YJB-T900), in which 50% (12/24) of the colonies produced diploid subpopulations (Fig. 1B and C). Thus, YJB-T900 has improved stability relative to the originally identified haploids (9), and the *Ac*/*Ds-NAT1* strains were consistently stable haploids and, like YJB-T900, exhibited improved growth relative to the originally isolated haploids (9) (Fig. 1A), making them ideal for transposon mutagenesis and the detection of recessive mutations.

### Preparation and characterization of large-scale *Ds*-*NAT1* insertion libraries.

Induction of the transposase (AcTPase4xCa) on maltose catalyzed excision of the *Ds*-*NAT1* transposon, thereby restoring *ADE2* expression and a shift from red (Ade negative [Ade^−^]) to white (Ade-positive [Ade^+^]) colonies (Fig. 1E). We generated libraries of C. albicans transposon (*Ca*Tn) mutants and passaged them twice to select for cells that had undergone *Ds-NAT1* excision and reintegration. Flow cytometry of DNA content and PCR of the *ADE2* locus of 96 Ade^+^ colonies (Fig. 1D) detected only those haploids that had undergone excision and reintegration.

Targeted sequencing identified *Ca*Tn insertions within the 11 libraries with the highest transposition frequencies (among 25 libraries initially prepared). Alignment of the resulting data (∼12 M to ∼32 M reads per library) (Table 1) with the reference strain sequence, SC5314 Assembly 22 haplotype A, identified the insertion sites relative to all annotated open reading frames (ORFs), functional RNAs, or transposons (see Table S1 in the supplemental material). Consistently, ∼one-third of the insertion sites (hits) were within annotated features and two-thirds fell in intergenic regions, with the proportion of annotated features that contained hits ranging from 55.5% in library 10 to 89.7% in library 3, yielding ∼2 to 14 hits per annotated feature (Table 1). Subsequent analysis focused on data combined from the three libraries (3, 7, and 11), each of which had an average of more than 1 hit/100 bp and approximately ≥10 hits per genomic feature (ORFs, noncoding RNAs, etc.). Together, these three libraries had ∼600,000 unique *Ds-NAT1* insertions, with 33.2% of them hitting within annotated features and 66.8% of them in intergenic regions, on the CGD (38). A total of ∼95% of the annotated features included at least 1 *Ds-NAT1* insertion site.

**TABLE 1:** Transposition library sequence quantification

Library^*a*^	Total no. of reads (×10^6^)	Total no. of hits (×10^3^)	Mean no. of hits/100 bp	% of hits in features	% of intergenic hits	% of features per hit	Mean no. of hits per feature	Mean no. of reads per feature (×10^3^)	Mean no. of reads per hit (×10^3^)	Mean no. of reads per hit in feature (×10^3^)
1	26.7	23.8	0.17	38.18	61.82	62.46	2.2	2.2	1.1	1
2	12.4	25.2	0.18	35.3	64.7	60.63	2.2	1.1	0.5	0.5
3*	31.9	252.6	1.77	33.39	66.61	89.72	14.2	1.7	0.1	0.1
4	20.2	40.6	0.28	31.99	68.01	69.87	2.8	1.4	0.5	0.5
5	30.9	64.6	0.45	31.71	68.29	74.12	4.2	2.1	0.5	0.5
6	19.2	40.1	0.28	31.61	68.39	69.84	2.7	1.4	0.5	0.5
7*	28.8	169	1.18	33.26	66.74	85.85	9.9	1.6	0.2	0.2
8	26.6	37	0.26	31.07	68.93	63.9	2.7	1.8	0.7	0.7
9	31.3	28.1	0.20	37.25	62.75	65.47	2.4	2.4	1.1	1
10	22.8	27.9	0.20	28.96	71.04	55.54	2.2	1.8	0.8	0.8
11*	23.5	178.2	1.25	32.9	67.1	85.59	10.4	1.3	0.1	0.1

a Asterisk indicates three libraries pooled for subsequent analysis.

10.1128/mBio.02048-18.6 TABLE S1 Annotated features in C. albicans. Download Table S1, PDF file, 0.2 MB. Copyright © 2018 Shtifman Segal et al. 2018 Shtifman Segal et al. This content is distributed under the terms of the Creative Commons Attribution 4.0 International license.

Insertion sites exhibited a periodicity reminiscent of nucleosome occupancy. Comparison of log-read and hit counts on each chromosome for regions with lower and higher likelihoods of being occupied by a nucleosome revealed a consistent bias toward a higher frequency of insertions in regions with a lower likelihood of nucleosome occupancy (Table S2).

10.1128/mBio.02048-18.7 TABLE S2 Number of insertions and reads in regions with low and high likelihood of nucleosome occupancy. Download Table S2, PDF file, 0.02 MB. Copyright © 2018 Shtifman Segal et al. 2018 Shtifman Segal et al. This content is distributed under the terms of the Creative Commons Attribution 4.0 International license.

**Fig 2:**
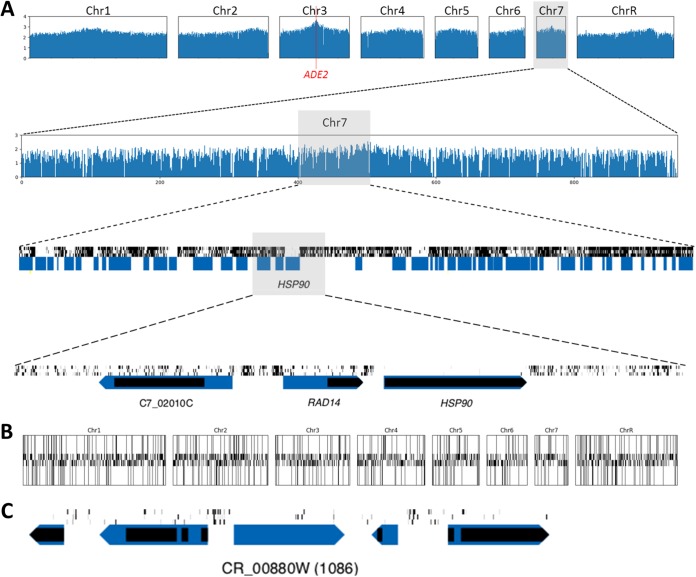
Maps of transposon insertions at different scales. (A) Distribution of insertion sites (hits) for the pooled library used in this study (libraries 3, 7, and 11). In the top two rows, the 8 C. albicans chromosomes and Chr7 are in 10,000-bp bins, the *y* axis scale is log10, and the *ADE2* gene is indicated by a red bar. In the lower two rows, 100-kb and 10-kb sections of Chr7 show three tracks of hit data in black, representing libraries 3, 7, and 11 (from top to bottom). Blue rectangles, predicted ORFs; black rectangles, recognized domains from protein information in CGD (38). The arrow points to the 3′ end of the gene. (B) Distribution of predicted essential and nonessential genes across the chromosomes. Essential genes are indicated with tall bars; nonessential genes are indicated with short bars; bars above and below the central axis represent genes transcribed on the Watson and Crick strands, respectively. (C) Example of low-hit region of CR_00880W gene.

**Fig 3:**
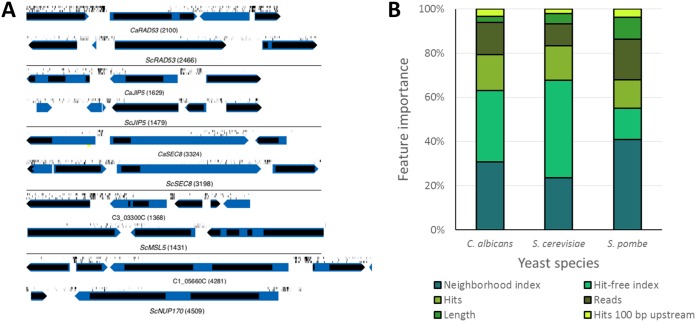
(A) Examples of genes with hits in the C-terminal portion of the coding sequence for C. albicans and S. cerevisiae orthologs. Note that patterns are similar although S. cerevisiae genes sometimes have insertions in the extreme N-terminal coding sequence that are not evident in C. albicans orthologs. Yellow bars below the genes indicate introns. Similar maps for the other libraries are found in Fig. S2A and maps of all ORFs are available in Dataset 9 at https://doi.org/10.6084/m9.figshare.c.4251182. (B) Relative feature importance contributions to the random forest classifiers for all three model yeasts.

*Ac*/*Ds* transposons tend to reinsert near the donor site at high frequency (previously reviewed [34, 39, 40]). A bias for insertion in regions close to the original *Ds-NAT1* insertion site at *ADE2* was evident, with the highest density of hits (number of insertion sites within an ORF) and reads (total number of sequences per hit or ORF) within ∼100 kb of *ADE2* on Chr3 (Fig. 2A; see also Fig. S1B, red bar). Despite this bias, genes very likely to be essential (those that sustained very few hits within the ORF and had many more hits detected in flanking intergenic regions [e.g., *HSP90*]) (Fig. 2A) were often evident. Yet intuitive visual analysis was not always sufficient to determine gene essentiality, especially in genome regions with lower hit density (see, e.g., Fig. 2C) as well as potential “domain-essential” genes that sustained hits only within a defined region of the coding sequence (see, e.g., Fig. 3A).

10.1128/mBio.02048-18.3 FIG S2 Hit maps of C. albicans and S. cerevisiae genes with conserved organization of essential domains, examples of misannotated C. albicans ORFs, and alignment of proteins that they predict. Download FIG S2, TIF file, 0.4 MB. Copyright © 2018 Shtifman Segal et al. 2018 Shtifman Segal et al. This content is distributed under the terms of the Creative Commons Attribution 4.0 International license.

### Prediction of gene essentiality on the basis of a machine learning approach.

To distinguish essential genes from nonessential ones in the transposon insertion data, we used a machine learning (ML) approach. Specifically, we constructed a random forest (RF) classifier with a set of features from the transposon data as follows: (i) the total number of hits per ORF, (ii) the total number of sequence reads per ORF, and (iii) the total number of hits within 100 bp 5′ to the ORF, as well as (iv) the ORF length, (v) the “neighborhood index” (the total number of hits per ORF normalized for the hits in surrounding intergenic sequences), and (vi) the longest hit-free region (normalized length of the largest ORF interval without hits) (Table 2). Training sets of presumed essential and nonessential genes were used to train a gene essentiality predictor that uses feature-based decision rules. Similar predictors were constructed for S. cerevisiae and S. pombe. Details are provided in Materials and Methods.

**TABLE 2:** Input features for the machine learning classifier

Feature	Definition
Hits	No. of insertion sites within the ORF
Reads	No. of reads within the ORF
Hits in promoter	No. of hits within 100 bp upstream of ORF start codon
ORF length	Total length of ORF coding sequence (intron-free)
Insertion index^*a*^	No. of hits in the ORF divided by ORF length
Noncoding window^*a*^	Noncoding sequence (including introns) within 10 kb up- and downstream of ORF
Neighborhood index (NI)	Insertion index normalized to the noncoding window (hits divided by length)
Hit-free interval (HFI)	Length of longest insertion-free interval divided by ORF length

a These features were input indirectly to calculate NI and HFI.

**Fig 4:**
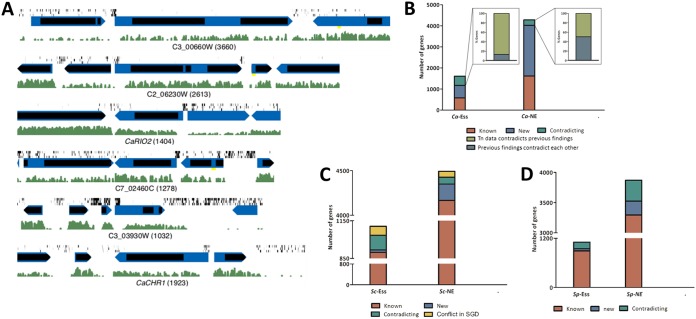
(A) Examples of genes with misannotated start codon positions. Symbols are as described in the Fig. 3 legend. Green histograms illustrate RNAseq expression levels (48). (B) Proportions of essential and nonessential genes compared to prior information are indicated. CaTn-Ess and CaTn-NE genes, as indicated. Red, genes with prior data indicating essentiality; blue, genes that were not previously tested for essentiality; green, genes with contradictory data. Dark green, genes with contradictory data prior to this study; light green, genes for which CaTn predictions contradicted at least one other report. (C) *Sc*Tn-Ess and *Sc*Tn-NE genes, as indicated. In yellow are genes that had conflicting annotations in SGD. (D) *Sp*Tn-Ess and *Sp*Tn-NE genes, as indicated.

We assembled training sets of C. albicans genes (see Dataset 1A at https://doi.org/10.6084/m9.figshare.c.4251182) on the basis of two major assumptions: (i) that most of the 697 ORFs with essential orthologs in both S. cerevisiae and S. pombe (which diverged from each other over 300 million years ago [MYA] [41]) were likely to be essential in C. albicans and (ii) that the 759 ORFs that had been deleted, which included genes with and genes without orthologs in the model yeasts, were unlikely to represent essential genes. These sets were further filtered by manual inspection (see Materials and Methods). The resulting sets of genes were used to train a random forest classifier with 5-fold cross validation which showed that the predictions exhibited high accuracy (area under the receiver operating characteristic curve [AUC], 0.997) (Fig. S2C). A threshold of 0.8 yielded a false-positive rate (FPR) of 1% and a true-positive rate (TPR) of 92% (Table 3) and identified 1,610 C. albicans essential (*Ca*Tn-Ess) and 4,383 nonessential (*Ca*Tn-NE) genes among the 5,893 ORFs (see Dataset 2A at https://doi.org/10.6084/m9.figshare.c.4251182). This provided first-time data for a total of 3,697 genes (1,033 *Ca*Tn-Ess and 2,664 *Ca*Tn-NE) (Fig. 4B). The *Ca*Tn-Ess genes and the *Ca*Tn-NE genes were distributed relatively randomly across the 8 C. albicans chromosomes (Fig. 2B).

**TABLE 3:** Cross-validation AUCs and thresholds chosen for prediction in each organism

Organism and training data from:	Cross validation AUC	Threshold	FPR^*a*^	TPR^*b*^
C. albicans	0.997	0.8	0.01	0.92
S. cerevisiae	0.989	0.67	0.02	0.89
S. pombe	0.966	0.62	0.04	0.80

a False-positive rate.

b True-positive rate.

To assess the reliability of the ML approach, similar analyses were performed with *in vivo* transposon insertion data for S. cerevisiae (34) and S. pombe (35), with training sets chosen from genes found to be essential or nonessential in deletion analyses in that organism (42, 43). The resulting accuracies (as reported by AUCs) were very high (Table 3).

The feature importance with respect to predicting gene essentiality with the random forest classifier differed somewhat between the three yeasts (Fig. 3B). The neighborhood index was the strongest predictor in C. albicans and S. cerevisiae. The “insertion-free region” was the most powerful predictor in S. pombe, where *Hermes* insertions were more evenly distributed, and was more important in C. albicans than in S. cerevisiae, possibly because S. cerevisiae tolerated insertions into interdomain regions and C. albicans did not. Not surprisingly, the nominal number of hits and number of reads per ORF also contributed substantially to the predictions, while ORF length and the number of hits within the 100 nucleotides (nt) 5′ to the start codon made only small contributions to predictions of essentiality in all three yeasts. Nonetheless, while ∼46% of the 1,610 *Ca*TN-Ess genes had no hits in the 100-nt 5′ untranslated region (5′UTR), only ∼9% of the *Ca*Tn-NE group had none, suggesting that this feature has some predictive power in C. albicans. Importantly, the feature set used was sufficiently robust to allow high-accuracy predictions across all three yeasts (Table 3). Furthermore, using just the 66 “CGD essential” genes (see Dataset 3 at https://doi.org/10.6084/m9.figshare.c.4251182) together with the original training set of deleted C. albicans genes, the results were also quite strong (AUC 0.92). This suggests that the training set of known essential and nonessential genes does not need to be large and that criteria for hit patterns established for these three yeasts may be sufficient to predict essentiality in other organisms.

### Comparison of deletion results (Δ) and transposon (Tn) predictions.

To test the accuracy of the ML classifier, we used data from either the S. cerevisiae SATAY (mini*Ds*) data (34) or the S. pombe Hermes transposon data (35) (Table 3) together with training sets from comprehensive deletion studies in the same organism (42, 43) and compared the ML predictions (see Dataset 2B and C at https://doi.org/10.6084/m9.figshare.c.4251182) to the genome-wide deletion study conclusions (*Sc*Δ and *Sp*Δ). The *Sc*Tn study (34) analyzed by ML predicted 1,106 essential genes (there are 975 *ScΔ*-Ess genes in SGD). There was agreement for 92% (898) *Sc*-Ess genes and 98% (4166) *Sc*-NE genes (Fig. 4C). The *Sp*Tn study (35) analyzed by ML predicted 1,106 *Sp*Tn-Ess genes (there are 1,241 *SpΔ*-Ess genes in PomBase), with agreement for 72% (895) *Sp*-Ess genes and 95% (3,293) *Sp*-NE genes (similar to the predicted TP rate of 0.80) (Fig. 4D).

Disagreement between the deletion and transposon data can be due to the presence of secondary suppressors (44) and other issues in deletion strains (35), to conditional essentiality (45), and to strain-specific effects (11, 45, 46), as well as to the difficulty encountered in identifying all of the domain-essential genes (44) (see, e.g., Fig. 3A; more detail is provided below and in Fig. S4A).

10.1128/mBio.02048-18.5 FIG S4 Specific examples of C. albicans genes and their paralogs in S. cerevisiae and/or S. pombe. Download FIG S4, TIF file, 0.4 MB. Copyright © 2018 Shtifman Segal et al. 2018 Shtifman Segal et al. This content is distributed under the terms of the Creative Commons Attribution 4.0 International license.

### Domain-essential genes.

Domain-essential genes have many transposon insertions within a portion of the ORF and were defined in SATAY as having no hits in a >400-bp domain (Fig. 3A) (34). For the ML predictions, we defined a “hit-free interval length” feature as the longest region without hits, divided by the ORF length (Table 2). In C. albicans, unlike in S. cerevisiae, hit-free regions were evident in C-terminal coding regions and not in the N termini. For example, *CaRAD53*, a gene involved in DNA damage responses and filamentous growth in C. albicans (47), had no insertions in the first 1,402 bp of the coding sequence, which is predicted to include a protein kinase-like domain. This suggests that the kinase domain is likely important, or essential, for *RAD53* function and that the C-terminal region may be dispensable (Fig. 3A). Similar domain patterns were seen for *JIP5*, *SEC8*, *and MSL5* (34) (Fig. 3A). Domain-essential ORFs were found in all three yeast species on the basis of the Tn insertion patterns, and in a few ORFs, domain insertion patterns were conserved (e.g., Fig. S2A).

### Refinement of transcription and translation start sites for several genes.

Unexpectedly, several genes appeared to be essential (very few hits within the ORF) and yet had high levels of insertions immediately upstream to and downstream of the start codon of the presumed ORF (Fig. 4A). In some cases, the S. cerevisiae ortholog (e.g, C7_02460C/*ScNPA3*) was essential and predicted to encode a shorter protein (Fig. S2C). Furthermore, in this example, data from transcriptome sequencing (RNAseq) (48) suggested that the transcription start site was 112 bp 3′ to the initiation codon (Fig. S2B) and that the predicted proteins aligned well from the downstream ATG codon (Fig. S2C). This demonstrates that transposon analysis can contribute to the identification of coding sequence boundaries.

### “Core essential” and “core nonessential” genes.

**Fig 5:**
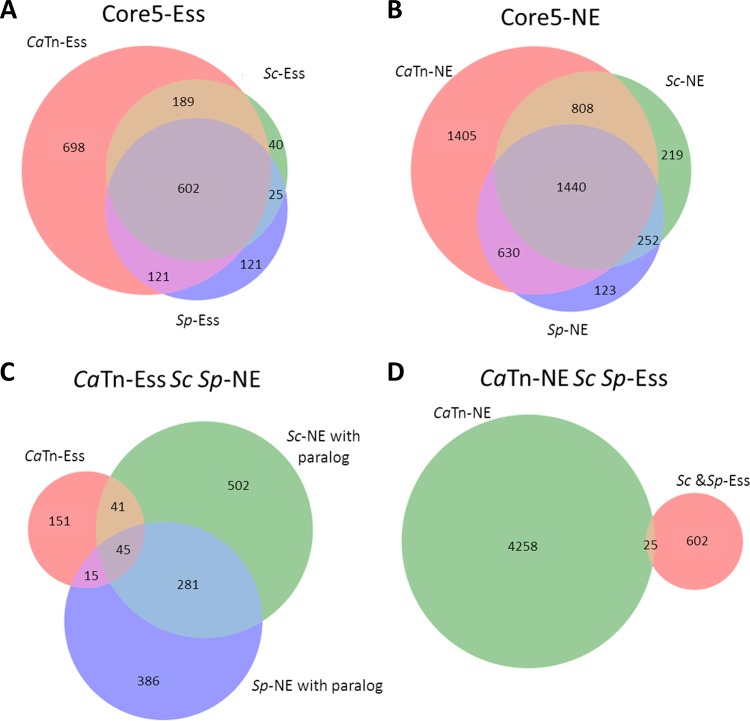
Venn diagrams of the intersections of (A) Core5 essential genes; (B) Core5 nonessential genes; (C) S. cerevisiae and S. pombe paralogs with *Ca*TnEss, *ScSp*NE group; and (D) *Ca*TnEss versus *Sc*Ess and *Sp*Ess.

Overall, 694 genes were essential in all three Tn studies (*Sc*Tn/*Sp*Tn/*Ca*Tn) and 602 were essential in all 5 deletion and Tn studies (*Sc*Δ/*Sp*Δ/*Sc*Tn/*Sp*Tn/*Ca*Tn) (Core5-Ess) (Fig. 5A). Interestingly, 17 Core5 genes were not essential in a wild-type S. cerevisiae strain (Sigma 1278 b) (11), which highlights several notable points. First, as noted in several previous studies (11, 49, 50), the essentiality of some genes is strain background specific even within a given species. Second, any specific C. albicans haploid strain is likely is be carrying deleterious alleles that are recessive in the heterozygous diploid parent (9), consistent with the lower growth rates of haploids (even those that are stable) compared to heterozygous diploids (Fig. 1A). We assume that deleterious recessive alleles with no phenotype in diploids may have epistatic effects on a subset of genes; accordingly, some genes may appear essential in a haploid derivative of a wild-type heterozygous diploid strain. Third, ML provides a statistical inference; thus, a small proportion of false-positive and false-negative predictions are expected. The confidence score (see below) is designed to reduce this uncertainty further.

**Fig 6:**
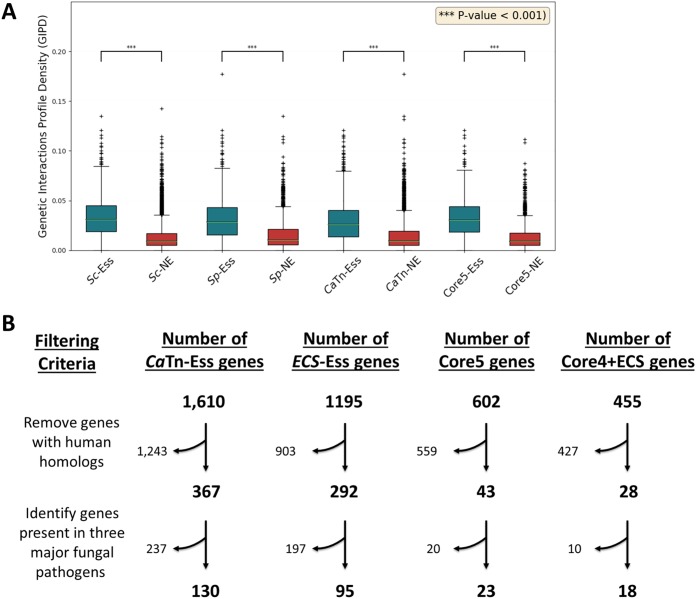
Genetic interaction degree and number of essential genes conserved in pathogenic fungi and not in humans. (A) Genetic interaction degree, a measure of interactivity of C. albicans genes in experiments performed using SGA software (see Materials and Methods) plotted for essential (Ess) and nonessential (NE) genes/orthologs in C. albicans (*Ca*Tn) and S. cerevisiae (*ScΔ* and *Sc*Tn) and from S. pombe analyses (*SpΔ* and *Sp*Tn) as well as from the Core5 analyses (*Ca*Tn, *ScΔ*, *Sc*Tn, *SpΔ*, and *Sp*Tn). The *y* axis data represent the number of gene interactions normalized by the number of observations (GIPD) (46). The statistical significance of results of comparisons between Ess and NE genes was obtained using the Wilcoxon rank sum test (***, *P* < 0.001). (B) Four groups of essential genes of *Ca*Tn-Ess and Core5-Ess as described in the Fig. 5 legend as well as of genes of *Ca-*ECS and Core5-ECS-Ess (after filtering was performed using an essentiality confidence score) were then filtered for those without human homologs and then for those with homologs in the other three major pathogenic yeasts (A. fumigatus, C. neoformans, and H. capsulatum).

The majority of the core essential genes were enriched for fundamental eukaryotic processes such as RNA metabolism, regulation, organelle organization, ribosome biogenesis, and cell cycle (Fig. S3). In addition, the *Sc*Tn-Ess genes had a higher degree of genetic interaction profile density (GIPD)—representing the number of synthetic genetic interactions for a given gene divided by the number of interactions tested (51, 52) (Fig. 6A). Notably, this relationship held true for essential versus nonessential orthologs of those genes in S. pombe and C. albicans as well (Fig. 6A).

10.1128/mBio.02048-18.4 FIG S3 Gene ontology (GO) term analysis of Core5 essential genes and Core5 nonessential genes. Download FIG S3, TIF file, 0.2 MB. Copyright © 2018 Shtifman Segal et al. 2018 Shtifman Segal et al. This content is distributed under the terms of the Creative Commons Attribution 4.0 International license.

### Orthologs essential in C. albicans and not essential in both S. cerevisiae and S. pombe.

Among the genes with orthologs in all three yeasts, 252 were essential in C. albicans (*Ca*Tn-Ess) and not essential in the two model yeasts via both deletion and Tn studies (Core4-NE) (Fig. 5; see also Dataset 2D at https://doi.org/10.6084/m9.figshare.c.4251182). In S. cerevisiae, remnants of the whole-genome duplication provide redundancy/backup functions under some conditions and thus may not be essential individually, whereas their single-copy orthologs in C. albicans are essential (53, 54). For example, in S. cerevisiae, *ScBDF1* and *ScBDF2* are each dispensable and yet dependent upon the presence of the other (55). The single gene *CaBDF1* was found to be essential both in CaTn analysis (see Dataset 2A at https://doi.org/10.6084/m9.figshare.c.4251182) and in classical deletion studies (56). Indeed, 101 of these 252 genes are found in single-copy form in C. albicans and have paralogs in at least one of the two model yeasts (57–59) (Fig. 5C). The remaining 151 genes that are essential in C. albicans and have no paralog in the model yeasts (Fig. 5C) were enriched for genes important for ATP synthesis via respiration and mitochondrial components (see Dataset 4 at https://doi.org/10.6084/m9.figshare.c.4251182), which highlights the rewiring of respiration and mitochondrial translation in C. albicans relative to the model yeasts (60). Several C. albicans genes involved in cell cycle progression were essential in C. albicans and nonessential in S. cerevisiae or vice versa (Fig. S4B).

Also of interest are obvious differences between insertion site frequencies in the three yeasts for *MET6* and *CLN3*, which are *Ca*Tn-Ess and *Sc*Tn-NE (Fig. S4C and D). *MET6* and *CLN3* were also shown to be essential in classical C. albicans deletion studies (61–63), suggesting that both gene products participate in processes that have diverged significantly in humans, making it a potential target for antifungal drugs (discussed below).

Among the genes essential in both C. albicans and S. cerevisiae, but not in S. pombe, were three septins essential for bud neck function (*CDC11*, *CDC12 and CDC3*), a process very different in fission yeast than in the two budding yeasts, and several components of the DASH kinetochore complex that is present in S. cerevisiae and C. albicans but is not conserved in S. pombe (see Fig. S4E and Text S1 in the supplemental material). Genes essential in both C. albicans and S. pombe but not S. cerevisiae included those corresponding to a range of functions; an example is CR_01480W/AIM10/SPBC24C6.03, which encodes a mitochondrial tRNA synthesis and affects the stability of the mitochondrial genome and is dispensable in S. cerevisiae but essential in C. albicans and S. pombe.

10.1128/mBio.02048-18.1 TEXT S1 Expanded supplemental figure legends with references. Download Text S1, DOCX file, 0.1 MB. Copyright © 2018 Shtifman Segal et al. 2018 Shtifman Segal et al. This content is distributed under the terms of the Creative Commons Attribution 4.0 International license.

### *Candida*-specific essential genes.

Comparisons among the three yeasts above necessarily involved analysis of genes with orthologs in at least two of the three yeasts. In addition to the essential genes that had orthologs in S. cerevisiae and/or S. pombe, there were 113 *Ca*Tn-Ess genes with no strict ortholog in either S. cerevisiae or S. pombe (38). Of these, 18 had sufficient similarity to S. cerevisiae genes to be annotated accordingly; 3 of those 18 were most similar to *ScHSK3*, *ScDUO1*, and *ScSPC34*, which encode components of the DASH complex (the outer kinetochore complex) that are essential in S. cerevisiae and wild-type C. albicans (64); these genes diverged rapidly and are not conserved in S. pombe, as noted above (65).

Of the 113 C. albicans essential genes with no homology or similarity to S. cerevisiae or S. pombe genes, 17 have no obvious orthologs among the two model yeasts or among the CUG clade species (see Dataset 5 at https://doi.org/10.6084/m9.figshare.c.4251182). None of them have been characterized directly, although transcripts have been detected for at least three of them under some growth conditions (48). By contrast, 55 of the CaTn-Ess genes had clear orthologs in all six pathogenic species related to C. albicans (see Dataset 5 at https://doi.org/10.6084/m9.figshare.c.4251182). The conservation of these genes supports the idea that they are important for the survival of the CUG group of fungi. It also suggests that future work should focus on the functions of these genes, as they have the potential to be clade-specific targets of antifungal therapies.

### Orthologs not essential in C. albicans but essential in both model yeasts.

Of the 627 genes found to be essential in all deletion and transposon studies in the two model yeasts (*Sc*Δ-Ess/*Sc*Tn-Ess/*Sp*Δ-Ess/*Sp*Tn-Ess genes), 4% (25 genes) were predicted to be nonessential in C. albicans (Fig. 5D). Two of these (C7_02460C and C2_06230W) had misannotation of the start codon (Fig. 4A), and six had large insertion-free domains (Fig. S4F), suggesting that they could be domain essential. Gene ontology (GO) term analysis of these CaTn-NE/*Sc*-Ess and *Sp*-Ess genes is enriched in mRNA processing (false-discovery-rate [FDR]-corrected *P* value, 5.82e−06) and U2-type spliceosomes component (FDR-corrected *P* value, 1.5e−6).

### Consistency among deletion, repression, and transposon insertion studies.

Prior to this study, 2 or more studies disagreed about essentiality for at least 190 (16%) of 1,183 genes, with new *Ca*Tn information for 2,996 genes with no prior experimental information; ∼13% (58/440 *Ca*Tn-Ess genes) correspond to at least one contradictory study (Fig. 4B). This highlights the difficulty in reaching definitive conclusions for every last gene.

To address this ambiguity, we calculated an essentiality confidence score (ECS) that captures available C. albicans essentiality information from deletion and repression data and Ca*Tn* studies (11–14, 19). The data from each study were considered equally (+1 for essential, −1 for nonessential), and the sum of their scores (net essentiality) for each gene was used with a logistic function to predict the likelihood of essentiality (using a 0-to-1 scale) (see Dataset 6 at https://doi.org/10.6084/m9.figshare.c.4251182; details in Materials and Methods). A caveat with respect to the ECS is that it assigns equal weight to each type of study. While the scheme can be generalized to learn individual weights from the different studies, we chose a simple scheme in order to avoid the potential biases that can stem from imperfect training data.

In total, the ECS identified 346/5,893 genes that were not clearly essential or nonessential (ECS = 0.5). Among these were primarily genes with two different outcomes (see Dataset 6 at https://doi.org/10.6084/m9.figshare.c.4251182). An interesting example is the riboflavin synthesis pathway in S. cerevisiae that requires *RIB1*, RIB7, *RIB3*, *RIB4*, *RIB5*, *FMN1*, and *FAD1* (66), which have been shown to be essential in deletion and/or Tn studies, as are the S. pombe orthologs (Table S3; see also Dataset 2D at https://doi.org/10.6084/m9.figshare.c.4251182). Six of these (*RIB1*, *RIB3*, *RIB4*, *RIB7*, *FMN1*, and *FAD1*) were *Ca*Tn-Ess, and yet four (*FMN1*, *RIB7*, *RIB3*, and *RIB4*) were nonessential by repression analysis (19); thus, their ECS was 0.5 and their essentiality remains “unknown” (Table S3). We suggest that mutations in the riboflavin biosynthesis pathway may cause extremely slow growth, leading to equivocal results that depend on the criteria used to determine essentiality and the medium used for growth studies.

10.1128/mBio.02048-18.8 TABLE S3 Riboflavin biosynthesis pathway analysis. Download Table S3, PDF file, 0.1 MB. Copyright © 2018 Shtifman Segal et al. 2018 Shtifman Segal et al. This content is distributed under the terms of the Creative Commons Attribution 4.0 International license.

### Essential genes conserved in fungi and not humans.

Essential genes are thought to be good targets for antifungal therapy because their inactivation should kill the pathogen (21). However, the similarities between fungi and their animal hosts present a challenge to the development of antifungal drugs. In theory, preferred antifungal targets should be essential genes without human homologs that are conserved among pathogenic fungi. Accordingly, we examined sets of essential genes, including the *Ca*Tn-Ess, ECS-Ess, and Core5-Ess genes and the Core4+ECS-Ess genes (essential in all 3 yeasts using the ECS filter for *Ca*-Ess genes) and then examined those without human homologs. Among these genes, we then identified the subset with homologs in the other three major human pathogens: Aspergillus fumigatus (Eurotiomycetes, Ascomycota), Cryptococcus neoformans (Tremellomycetes, Basidiomycota), and Histoplasma capsulatum (Eurotiomycetes, Ascomycota). We found 130 *Ca*Tn*-*Ess genes and 95 ECS-Ess and 23 Core5-Ess and 18 Core4+ECS genes that have homologs in the other three major fungal pathogens and that do not have human homologs (Fig. 6B; see also Dataset 7 at https://doi.org/10.6084/m9.figshare.c.4251182).

Among the *Ca*Tn-Ess and ECS-Ess genes were 52 genes whose products are mitochondrial and that are involved in ATP synthesis and/or mitochondrial membrane function, 4 of the 5 genes encoding ERMES complex components (*MDM10*, *MDM12*, *MDM34*, and *MMM1*), and 7 genes encoding kinetochore components [*SPC19*, *MTW1*, *ASK1*, *CaMAD1*, *CaSPC105*, *CaBIR1*(CR_05100W), and *CaKRR1*]. In addition, components of the riboflavin pathway did not have human homologs and had homologs in the other pathogenic fungi (see Dataset 5 at https://doi.org/10.6084/m9.figshare.c.4251182). We suggest that genes in these groups have the potential to be high-priority candidates for broad-spectrum antifungal drug design.

## DISCUSSION

This study leveraged a stable haploid isolate, a codon-optimized, inducible *Ac* transposase, strong selection of excision and reintegration events, and machine learning to double the number of predicted genes that are essential or nonessential for C. albicans survival under laboratory growth conditions. The ML classifier trained on the three yeasts studied here is highly accurate and has the potential to work across species. We also developed an essentiality confidence score that considers different types of mutation studies, thereby providing a useful perspective on the degree to which essentiality is conserved and, together with comparisons to predicted ORFs in other pathogenic fungi and in Homo sapiens, that identifies those essential genes that have the potential to be targets for antifungal drugs.

### Genome-wide *Ac*/*Ds* mutagenesis in C. albicans haploids.

We leveraged two critical resources—a highly stable C. albicans haploid strain that does not autodiploidize and *in vivo* transposition using a modified *Ac* transposase/*Ds-NAT1* two-element system. A strength of the *Ac*/*Ds* transposon is that, unlike Hermes or PiggyBac (67, 68), it does not have a strong insertion site preference either in maize or in other organisms, including S. cerevisiae (34, 37, 40, 69).

The *in vivo* transposition is extremely efficient: once a starting strain is engineered, no further transformation or homologous recombination steps are required. This is particularly useful for clinically relevant organisms where transformation and homologous recombination limit the transfer of deletion constructs to a new strain background (45, 70–73). *In vivo* transposition also obviates the inherent bias present when researchers select, and thereby limit, the sequences to be analyzed, e.g., regions between ORFs (e.g., misannotation of transcription start sites) (Fig. 4A) and potentially essential noncoding RNAs (Ca22chrRA: *n* = 1,421,741 to 1,424,356).

*Ds* insertion preferences were detected for the following three features of DNA: nucleosome occupancy, proximity to the excision site, and intergenic versus coding features. The nucleosome bias was weaker in C. albicans than in the SATAY system (34). This was likely due, at least in part, to differences in nucleosome occupancy between the diploid C. albicans strain used for nucleosome coverage data (74) and the haploid *CaTn* strain. Proximity to the initial site of excision was evident in our libraries and has been well documented in lower-throughput studies (39) and in SATAY (34), while it was far less evident when the site of *Ds* excision was on a plasmid. Unfortunately, autonomously replicating plasmids are not maintained well in C. albicans. We are currently constructing plasmids that may be useful for this purpose in the future (J. Berman, unpublished results).

### Applying machine learning to *in vivo* transposon insertion data.

Previous transposon studies relied on statistical models specific to the organism and transposon, which may be advantageous when specialized information can be uniquely captured (28, 75) but are not generalizable and may be prone to error by the nature of the model specialization required. Here we applied an ML approach, which is more general and easier to implement and has the distinct advantage of being able to integrate an arbitrary number of data features (hits, reads, neighborhood, etc.). Interestingly, the classifier and feature set chosen were powerful enough to achieve accurate predictions across organisms—for example, the classifier for S. pombe achieved an AUC of 0.985 in the C. albicans benchmark (Table 4), despite the different transposon used (Hermes).

**TABLE 4:** AUCs of across-species benchmarks

Species	Across-species benchmark AUC		
	C. albicans	S. cerevisiae	S. pombe
C. albicans		0.981	0.938
S. cerevisiae	0.993		0.942
S. pombe	0.985	0.97	

A disadvantage of a supervised ML approach is the requirement for a reliable training set – prior knowledge of the essentiality status of a considerable number of genes. For determinations of gene essentiality, especially in a nonmeiotic organism, no training set can be perfect, due to the presence of conditionally essential genes and strain-specific genes (3). Even model yeast data are subject to artefacts (35, 44). Nonetheless, annotations of homologs from relatively distant evolutionary relatives, as well as training on a smaller subset of ~70 Ess and NE genes, were sufficient for strong predictions. Coupled with the possibility of training the classifier on a training set from a different organism, this approach should be applicable for the analysis of *in vivo* transposon data from other non-model organisms.

### Similarities and differences in gene essentiality.

The Core5-Ess genes are primarily involved in central processes such as gene expression and cell cycle progression (see Fig. S3 in the supplemental material) and are more likely to be “hubs” (have a high number of genetic interactions) in synthetic genetic array analysis experiments (51) (Fig. 6A). This is consistent with the idea that essential genes are more frequently engaged in central processes that involve larger numbers of genetic partners than nonessential genes.

Genes nonessential in C. albicans and essential in both S. cerevisiae and S. pombe were U2-type spliceosome components (corrected *P* value, 1.5^−6^; false-discovery rate, 0.00%), despite the small number of predicted introns in C. albicans (361) and S. cerevisiae (273) relative to S. pombe (2,394). This is consistent with the loss of highly conserved snRNA binding proteins and changes in snRNA sequences within the spliceosome catalytic site in C. albicans relative to S. cerevisiae (76) and supports the idea that spliceosome components evolved rapidly in the hemiascomycete yeasts.

With SATAY, some ORFs were enriched for insertions within the N-terminal region of the coding sequence and caused gain-of-function mutations. This was not evident in either *Ca*Tn or *Sp*Tn data. We suggest that the larger size of both Hermes and the *Ds-NAT1* may be less permissive with respect to spurious transcription initiation events that occur with the smaller *Ds* used in SATAY (34).

The different types of data (e.g., deletions and repression and transposon insertions *in vitro* and *in vivo*) provide a more complete view of gene functions than any single study. The ECS logistic function brings the list of genes with unclear essentiality to less than 6% (see Dataset 6 at https://doi.org/10.6084/m9.figshare.c.4251182). Potential inaccuracies in the *in vivo* transposon approach have several sources. First, DNA in cells that grow slowly or have died can still be amplified and inflate estimates of Tn-NE genes. Second, essential domains likely differ in a gene-specific manner that defies definitive categorization. Third, ML provides a statistical measure of the essentiality likelihood dependent on the training set quality, which is clearly imperfect. Finally, some C. albicans genes could be conditionally essential because of specific alleles that are present or absent in the haploid strain relative to the parental heterozygous diploid. There are likely to be one or more deleterious alleles in the haploid haplotype (77) as evidenced by their reduced growth rate and virulence relative to SC5314, the heterozygous diploid strain. Such allele-specific interactions could be akin to epistatic synthetic genetic interactions between a deleted allele and another partially functional allele elsewhere in the genome. Other genes may be conditional if they rendered C. albicans more sensitive to nourseothricin, which was used to select for the *Ds-NAT1.* Future studies of a range of haploid strains with different transposon markers have the potential to address this issue.

### Identifying potential drug targets.

C. albicans represents a serious economic and health threat as a human pathogen (5), and the limited armamentarium of antifungal drugs is a major challenge. This work has identified genes essential in C. albicans and Core5-Ess genes that lack human homologs. One major group of these genes consists of components of the DASH kinetochore complex, emphasizing differences with human kinetochores (64). Importantly, 130 essential C. albicans genes have no human homologs but do have homologs in the other three major human fungal pathogens. We suggest that these 130 genes are of high priority as potential targets for antifungal drug design because they could target a larger set of fungal pathogens rather than be specific only to C. albicans or to the CUG clade (see Dataset 7 at https://doi.org/10.6084/m9.figshare.c.4251182). Notably, two of these genes, C1_01490W and C3_07550C, were important for infectivity in a mouse model of candidiasis (13, 78). C1_01490W encodes a plasma membrane protein (79) that is repressed by nitric oxide (80). Analysis of the repression collection using the systemic mouse model found 19 of the essential genes with no human homolog and with homologs in the 4 pathogens. These included several genes involved in the ergosterol biosynthesis pathway, a component of the DASH kinetochore complex, and *MET6.* They also include groups of genes enriched in mitochondrial membrane organization, drug metabolism, aromatic amino acid synthesis (*ARO1*, *ARO2*, and *ARO7*), and riboflavin biosynthesis (19). The riboflavin biosynthesis pathway is absent in mammals (81), and at least some of its components are essential in the three yeasts analyzed, suggesting that this pathway may be an interesting target for antifungals. Indeed, a recent study ranked the synthesis of riboflavin as being a rich source of antifungal targets (81).

### The potential of *in vivo* transposition approaches for other purposes.

With essential genes identified, the next steps include analyzing the enrichment and depletion of genes in the existing pooled library analyzed here for those nonessential genes that are depleted or enriched under different growth and stress conditions. Meta-analysis of the results will establish the regulatory and metabolic networks that represent relevant host niches.

Another application is pooled synthetic genetic array analysis (51), which is performed by inducing transposition in strains carrying one or more mutations of interest and which will facilitate the detection of genetic interactions at the genome scale in an unbiased and relatively rapid and cost-effective manner. In addition, modifications to the transposon (39) can be engineered to add fluorescent protein or epitope tags to identify genes encoding proteins with specific cellular localization or protein-protein interactions. Inserting strong promoters (“activation tagging”), repressors, DNA binding proteins (e.g., *lacI*, *TetR*, or C. albicans DNA binding domains) or sequences that tether the target gene product to a specific cell structure can provide new functional insights as well. I*n vivo* transposition also can guide domain structure-function studies, as elegantly demonstrated in SATAY (34).

In summary, analyzing large numbers of *in vivo* generated transposon mutants produced in a stable C. albicans haploid strain allows the rapid and efficient analysis of gene essentiality and the identification of potential antifungal drug targets. It also has the potential to greatly improve the amount and quality of phenotypic information available for studying non-model as well as model organisms.

## MATERIALS AND METHODS

### Plasmids and strains.

All C. albicans strains derived from strain YJB-T900 (GZY896; kindly provided by Guisheng Zeng and Yue Wang) and are listed in Table S4A in the supplemental material. YJB-T900 is a derivative of haploid XI (9), which was ultimately derived from laboratory strain SC5314. C. albicans was grown at 30°C in rich YPAD medium (1% yeast extract, 2% peptone, 2% glucose) under normal conditions and in YPAM medium (1% yeast extract, 2% peptone, 3% maltose) when *Ac*TPase4xCa was induced. Transformants of C. albicans were selected in synthetic complete medium (SDC) (0.17% yeast nitrogen base with ammonium sulfate [Formedium], 2% glucose) supplemented with a dropout mix containing amino and nucleic acids except adenine or uridine, depending on the auxotrophic requirement for the selection (82).

10.1128/mBio.02048-18.9 TABLE S4 Yeast strains and plasmids and primers used. Download Table S4, PDF file, 0.4 MB. Copyright © 2018 Shtifman Segal et al. 2018 Shtifman Segal et al. This content is distributed under the terms of the Creative Commons Attribution 4.0 International license.

All media were supplemented with uridine (80 µg ml^−1^) or adenine (40 µg ml^−1^) except when used for selection of *URA3* or *ADE2* transformants. For solid media, 2% Bacto agar was added. Nat^+^ transformants were selected by plating the transformation mix on YPAD medium and replica plating the following day onto YPAD plates supplemented with 400 µg nourseothricin ml^−1^ (Jena Bioscience, Jena, Germany). All C. albicans transformations were performed following the haploid electroporation protocol (83). Escherichia coli strain DH-5alpha (Bio-labs Ltd.), and standard media and methods (84) were used for plasmid manipulations.

Yeast genomic DNA was isolated according to a previously described method (85).

Strain YJB-T1792 was constructed by directly transforming yeast with a fragment containing the *Ac*TPase4xCa expression cassette together with a *URA3* marker and flanking sequences from the C. albicans *NEUT5L* locus (86) from *NaeI*-digested BJB-T135/pKM300 (37) into strain YJB-T900. To integrate *Ds-NAT1* into the *ADE2* promoter, strain YJB-T1792 was transformed with NotI-digested BJB-T133/pRK402 (Table S4B). The correct integration of both the *Ac-URA3* and *Ds-NAT1* insertions was verified by PCR amplification of genomic DNA using primers BP104 and BP161 for the *Ac-URA3* and primers BP117 and BP118, primers BP119 and BP120, and primers BP117 and BP120 for *Ds-NAT1*. Exact insertions were confirmed by Sanger sequencing of the amplified fragments generated with these primer sets. The final strain, YJB-T1081, which includes both *Ac-URA3* and *Ds-NAT1* in the *ADE2* promoter, produces red colonies.

### Ploidy verification by flow cytometry.

Flow cytometry was performed as described previously (9) using a MACSQuant flow cytometer (Miltenyi Biotec GmbH, Germany) and SYBR green (Lumiprobe) to stain DNA. Ploidy levels were determined relative to known diploid and haploid isolate data.

### Growth analysis.

Strains were grown in SDC in a 96-well microtiter plate, and absorbance at 600 nm was measured every 15 min with a Tecan Infinite F200 Pro (Tecan, Switzerland) plate reader for 24 h. Haploid YJB-T900 parent and YJB-T1792 (Ac-only strain) and YJB-T1081/YJB-T1082 (*Ac*/*Ds-NAT1* strain from same initial transformant) strains were grown in SDC medium and showed identical growth rates, which were lower than those of the diploid strain SC5314 and yet higher than those of haploid I (YJB12801) and haploid XI (YJB12881, which was the parent of YJB-T 900), haploid isolates previously studied (9) (Fig. 1A).

### Virulence tests in mice.

A total of 15 C57BL/6J female mice, 6 to 8 weeks old, were purchased from Charles River UK Limited and maintained for 1 week at the Medical Research Facility (MRF) at the University of Aberdeen before the experiments were performed.

Candida albicans strains SC5314, YJBT1792, and YJBT1082 were grown overnight in SD-Ura minimal medium. The cells were washed three times with sterile phosphate-buffered saline (PBS), and suspensions of 2 × 10^6^ cells/ml were made in sterile PBS. Five mice per group were randomly chosen for each *Candida* strain and injected with 100 µl of cell suspension (2 × 10^5^ cells/mice) via the tail vein. The mice in each group were housed together in single individually ventilated cages (IVCs) in category II room facility at the MRF. Food and water were provided to mice *ad libitum*.

The mice were monitored daily for 2 weeks postinfection. Body weights were recorded each day, and mice were checked twice daily for any signs of clinical illness per the clinical scoring sheet (see Text S1 in the supplemental material). Mice showing a 20% weight loss or a clinical illness score of 2.5 were culled immediately by cervical dislocation. All surviving mice were culled at the end of study period.

The experimental design and protocol were approved by the study plan team at the MRF, and the experiments were performed under U.K. Home Office project license 70/8073 (to Gordon Brown).

### Generation of insertion libraries.

A total of 10^9^ YJB-T 1081 cells were grown in 25 ml of freshly prepared YPAM medium for ∼20 to 24 h to induce transposition events. Cells were collected by centrifugation (5 min at 1,000 × *g*, 20°C), washed twice with double-distilled water (ddH_2_O), and plated on ∼500 9-cm-diameter plates containing 25 ml of SDC-Ade+nourseothricin. Colonies in which transposon excision repaired the *ADE2* gene appeared after 48 h. All colonies are then scraped off the plates using sterile ddH_2_O, pooled, washed, and frozen in 15% glycerol. To dilute any remaining Ade^−^ or dead cells, ∼10^9^ cells were inoculated in 25 ml SDC-Ade+nourseothricin medium for 48 h. The saturated culture was harvested by centrifugation (5 min, 1,000 × *g*), washed with sterile ddH_2_O, and the cell pellets were frozen (Fig. 1E).

### Fractionation with Percoll.

To further separate Ade^−^ cells from the pooled culture, we adopted a Percoll separation protocol (87). Percoll (GE17-0891-01) was diluted 9:1 (vol/vol) with 1.5 M NaCl. A Percoll gradient was formed using 10 ml of the Percoll solution in 15-ml tubes that were centrifuged at 13,000 rpm for 15 min at 20°C. Approximately 2 × 10^9^ cells were pelleted, resuspended in 1 ml Tris buffer (pH 7.5), overlaid onto the preformed gradient, and centrifuged at 400 × *g* for 60 min in a tabletop centrifuge equipped with a swinging bucket rotor (Thermo Instruments) at 20°C. White cells were collected (Fig. 1E), washed once in 40 ml Tris buffer (pH 7.5), pelleted, and resuspended in ddH_2_O, and then yeast genomic DNA was isolated (85) to produce the insertion libraries.

### Transposon insertion sequencing.

A 1-µg volume of genomic DNA from each insertion library was randomly sheared using a Covaris S2 device (Covaris Inc., Woburn, MA, USA). Single-end Illumina libraries were constructed by ligation of Illumina adapters (Table S4C) to sheared DNA. Enrichment of transposon/chromosomal junction regions was performed by PCR amplification with a 5′ biotinylated transposon enrichment primer (BP664) and adapter-specific PCR enrichment primer R_Tnseq_index (Table S4C). All the following transposon sequencing steps were performed as described previously (29, 30). The resulting transposon libraries were quantified on an Agilent 2200 TapeStation system using HS D1000 tape and sequenced using an Illumina NextSeq 500 system (Illumina, USA) high-output v2 kit. The resulting library insertion sequences are available at NCBI under project PRJNA490565 (https://www.ncbi.nlm.nih.gov/bioproject/PRJNA490565).

### Analysis of transposon libraries. (i) Insertion site mapping.

Of the 11 C. albicans AcDs transposition libraries, the three with two or more insertions per 100 bp (Table 2), libraries 3, 7, and 11, were used for further analysis. Each library’s sequence FASTQ files were processed with cutadapt (88) (version 1.9.1) to remove the leading transposon sequence from the reads and possible trailing Illumina adapter. Reads that did not have the leading transposon sequence were discarded. The remaining reads were mapped onto the C. albicans reference genome using bowtie2 (89) (version 2.2.9) (using the “–very-sensitive” global alignment setting), and the output SAM files were compressed into the BAM format using SAMtools (90) (version 1.3.1). Reads that received a mapping quality score below 20 (i.e., probability of more than 1% of alignment to another region in the genome) were discarded. If an insertion location was attested by reads from both strands, it was counted as two separate insertion events; otherwise, it was counted as a single insertion event. We also observed that high-read-number insertions often would have single-read insertions adjacent to them, differing by only one nucleotide. We deemed this a sequencing error and counted every pair of immediately adjacent insertions as a single insertion. Further in the analysis, insertion sites from the 3 libraries were pooled and treated as a single data set (though they are shown separately in the figures), maintaining the constraint that every base pair was allowed at most two insertions. Numbers of insertions in all of the libraries were determined (see Dataset 8 at https://doi.org/10.6084/m9.figshare.c.4251182).

For S. cerevisiae, we used already-mapped insertion libraries WildType1 and WildType2 (34) and pooled them into one data set for further analysis. For S. pombe, we used sequencing data from references 35 and 94 (accession no. SRA043841.1 and SRR327340) and performed the mapping against version ASM294v2.30 of the S. pombe genome as described above for C. albicans, with the additional step of removing trailing bases that had a sequence quality score below 20 from the sequence file before mapping.

### (ii) Selection of appropriate genes for analysis.

The assignment of hits to repetitive genomic regions is ambiguous. To find such regions, we simulated a FASTQ file representing all possible 131-bp-long reads from the C. albicans genome (the mode of read length distribution in the transposon libraries) and aligned it as described above. Consecutive regions with read alignments of mapping quality below 20 were ignored in further analysis, and genes that had more than 5% of such regions (142 genes overall) were discarded. For example, *TEF1* and *TEF2* (see Fig. S4G in the supplemental material) were excluded because their coding sequences are >85% identical. Additionally, in the case of C. albicans, some regions were not mapped at all, due either to deletions (e.g., *URA3* and *GAL1* were deleted during the construction of the strain) or to issues in sequencing. As in the case of the repetitive regions, we discarded 12 genes with >5% unmapped regions. Finally, for both training and prediction, only protein-coding genes were considered (marked as ORFs in CGD). Overall, 5,893 genes among 6,198 C. albicans ORFs and 6,620 annotated features in the CGD were used in the analysis (see Dataset 2A at https://doi.org/10.6084/m9.figshare.c.4251182).

A similar repetitive region analysis was performed for S. cerevisiae and S. pombe, with simulated reads with lengths of 75 and 40, respectively, matching the modes of the read lengths in their sequenced data sets (34, 35). Additionally, in S. cerevisiae, ORFs marked as “dubious” and ORFs that had no insertions in them and in a 20-kb window surrounding them were removed, resulting in 5,599 genes being used among 7,542 features (see Dataset 2B at https://doi.org/10.6084/m9.figshare.c.4251182). In S. pombe, 4,985 ORFs among 5,129 were used, 144 ORFs being excluded due to ≥5% genomic duplication (see Dataset 1C at https://doi.org/10.6084/m9.figshare.c.4251182).

### (iii) Construction of the training sets.

We constructed “gold standard” training sets of genes that were likely to be essential or nonessential. Genes that had orthologs in both S. cerevisiae and S. pombe and that were consistently marked as essential (i.e., without contradicting evidence) in both organisms were considered likely to be essential (697 genes overall). In contrast, genes that were successfully deleted in a number of high-throughput deletion studies: (12–14) were considered to be likely nonessentials (759 genes overall). Three genes (*CDC19*, *SGT1*, and *PWP1*) were present in both data sets and were discarded. Because the assumptions upon which the training set were imperfect, as not all functions may be conserved, some of the reported deletion mutants might have acquired suppressors (3) and some screens for essentiality might have used different growth conditions. Thus, all 759 genes were visually inspected by three independent observers and we discarded 66 genes that were designated to be clear outliers by all of the observers. The complete training set and those genes that were manually excluded from it were determined (see Dataset 1A at https://doi.org/10.6084/m9.figshare.c.4251182).

For S. cerevisiae and S. pombe training sets, we used a similar construction method (697 essentials in S. cerevisiae and 689 in S. pombe), with the exception that the nonessential training set was constructed from orthologs in both S. cerevisiae and S. pombe that were marked in both by deletion studies (1,777 nonessentials in S. cerevisiae and 1,620 in S. pombe). Outliers were manually excluded as described above (in S. cerevisiae, 6 were discarded as false positives and 32 as false negatives; in S. pombe, 46 were discarded as false positives and 62 were discarded as false negatives).

### (iv) Construction of a gene essentiality predictor.

We used the implementation of the random forest classifier (91) in the scikit-learn library (92) (version 0.18.1) with the default parameters. The classifier features are listed in Table 2. The classification quality was measured using the area under the receiver operating characteristic curve (AUC; values ranged from 0 to 1), which describes the sensitivity versus the specificity of the predictions (Fig. S1C). For all organisms, the AUCs were high, with the AUC value for C. albicans being an almost perfect 0.997, greatly outperforming the random expectation AUC of 0.5. For setting the classification threshold, we used a 5-fold cross validation setting. We chose a false-positive rate of 0.9%, yielding a true-positive rate of 92% and a threshold of 0.8 (Table 3). To assess the possibility of cross-organism predictions, we tested each classifier on training sets and features from the other two organisms (see Dataset 1B and C at https://doi.org/10.6084/m9.figshare.c.4251182). Feature importance was evaluated as the mean decrease in impurity (93) as reported by scikit-learn.

All of the code and required dependencies are available at https://github.com/berman-lab/transposon-pipeline.

### Determining the essentiality confidence score (ECS).

To capture the contribution from all studies, we first assigned values to each gene in each data set, for each study independently, with scores of +1 for essential, 0 for no data, and −1 for nonessential, on the basis of the reported study results. This was done for deletion studies (11–14), for the newer repression study (19), and for the Tn study, where we assigned discrete “RF verdict” scores of +1 and −1 on the basis of the prediction verdict (RF score of ≥0.80, Ess; RF score of <0.80, NE) as described above.

The “net essentiality score” was then determined as the sum of all the values from all the other studies. The net essentiality scores ranged from +2 to −6 in discrete integer steps. (There are more deletion experiments, which can give scores of only −1 each, relative to the repression and *Ca*Tn experiments, which can give +1 and −1 results).

We then determined the essentiality confidence score (ECS) by applying a logistic function to the net essentiality score as follows:

$$\text{ECS}=\frac{1}{1+e^{-aX_{i}}}$$

where *a* = 1.55 was determined to achieve a value of >0.95 when *x* = 2 and a value of >0.99 when *x* = 3 and where *X_i_* is the net essentiality score (sum of all studies) for a given gene. The resulting ECS range was 0 ≤ *X_i_* ≤ 1 for each gene.

### Nucleosome bias analysis.

The likelihood of mononucleosome occupancy in C. albicans was determined by mapping read depth from micrococcal nuclease experiments (74, 94) (accession no. SRR059732) as a measure of nucleosome occupancy likelihood (where higher numbers of reads correspond to a higher likelihood of nucleosome occupancy). For each chromosome, the median read depth was used to separate it into regions of high and low nucleosome occupancy likelihood. We then compared the numbers of TnSeq log reads and numbers of hits between the two region types for each chromosome in each high-insertion density library. Results are shown in Table S2 (Mann-Whitney U test yielded *P* value for every comparison, <10^−6^).

### Sequences and annotations.

For C. albicans, the reference genome was haplotype A of Assembly 22, version s07-m01-r08 (38). For S. cerevisiae, the reference genome was R64-2-1 (95). For S. pombe, the reference genome was ASM294v2.30 (96). C. albicans ortholog and protein domain annotations were taken from the CGD (38). The S. cerevisiae feature annotations were downloaded from the SGD website (42). Essential and nonessential genes were called by collecting all phenotype annotations on the website and using only those that were annotated exclusively as either essential or nonessential. The S. pombe feature and essentiality annotations were downloaded from PomBase (43, 97).

Note that the specific transposons used in the three yeasts differed in a number of ways. The *Ds* used for C. albicans is 1,812 bp and includes the *Nat1* ORF; the mini-*Ds* for SATAY is only ∼600 bp (34); and the Hermes transposon for S. pombe was ∼1,000 bp in length and included the ∼1,500-bp kanMX6 ORF (35). Notably, insertion of the mini-*Ds* in the SATAY study resulted in both loss-of-function and gain-of-function mutations (34), while only loss-of-function mutations were evident for the C. albicans *Ds-NAT1* and the S. pombe Hermes insertion mutants.

### Analysis of genetic interaction density.

Genetic interactions of S. cerevisiae genes were obtained using synthetic genetic array (51) and kindly provided by B. VanderSluis. For each gene, the number of interactions was normalized by the number of observations/experiments in which that gene was measured, which provided the GIPD score. GIPD scores for multiple alleles of the same gene were averaged. Negative genetic interactions were found to be more functionally informative (51). “Stringent” negative GIPD (nsGIPD) scores (98) were selected for further analysis in C. albicans and S. pombe orthologs, filtered by the use of the genetic threshold described by Costanzo et al (51). Essential and nonessential gene populations in each organism were compared using the Wilcoxon rank sum test.

### Examining the conservation between human genes and fungal pathogens.

To determine the number of *Ca*Tn-Ess genes with homologs in humans or the major human pathogens, we conducted individual searches with each essential C. albicans gene for a homologous gene in the relevant proteome using BLASTP from NCBI’s BLAST+, version 2.3.0 (99), and an expectation value threshold of 1e−3 as recommended for searches for homologous sequences (100). We then compared proteins encoded by the *Ca*Tn-Ess genes to the proteomes of Aspergillus fumigatus af293 (Eurotiomycetes, Ascomycota), Cryptococcus neoformans H99 (Tremellomycetes, Basidiomycota), and Histoplasma capsulatum H88 (Eurotiomycetes, Ascomycota) using proteome data for the fungi obtained from FungiDB (http://fungidb.org/fungidb/) release 38 and human proteome data from the NCBI (Human Genome Assembly GRCh38.p12). To determine homologs, we used the same approach as described for human homologs using BLASTP from NCBI’s BLAST+, version 2.3.0 (100), using an expectation value threshold of 1e−3. Of course, we cannot rule out the possibility that distant orthologs were not detected with the stringent sequence similarities used here.

### Data availability.

All of the code and required dependencies for analysis of the TnSeq data are available at https://github.com/berman-lab/transposon-pipeline.

Library insertion sequences are available at NCBI under project PRJNA490565 (https://www.ncbi.nlm.nih.gov/bioproject/PRJNA490565). Datasets S1 through S9 are available at https://doi.org/10.6084/m9.figshare.c.4251182.
