# Supplementary figures and images for: Gene Essentiality Analyzed by In Vivo Transposon Mutagenesis and Machine Learning in a Stable Haploid Isolate of Candida albicans
Source: mBio. 2018 Oct 30;9(5):e02048-18. doi: 10.1128/mBio.02048-18 (PMC6212825; doi:10.1128/mBio.02048-18)

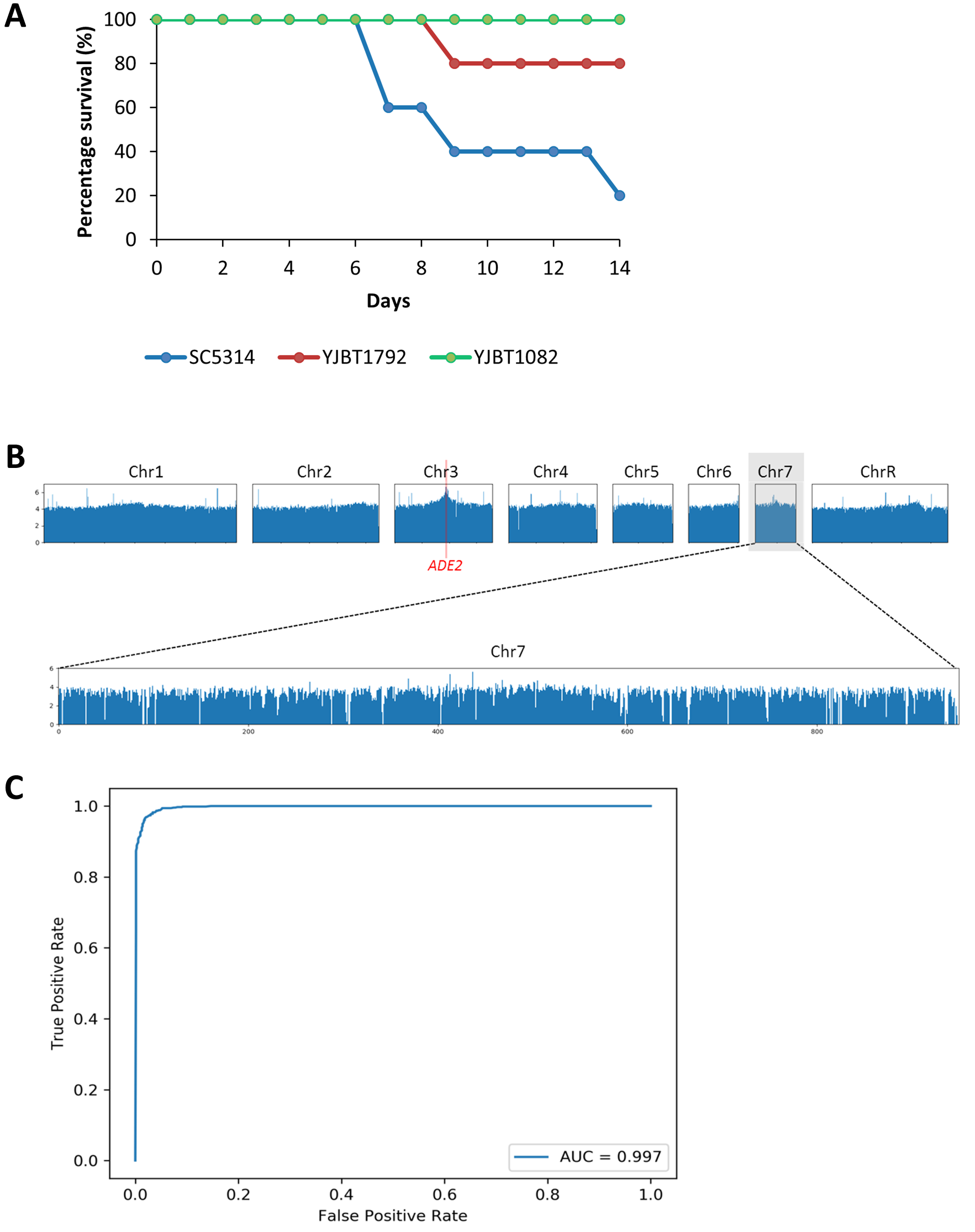

Supplement: FIG S1 [file mbo005184136sf1.tif]

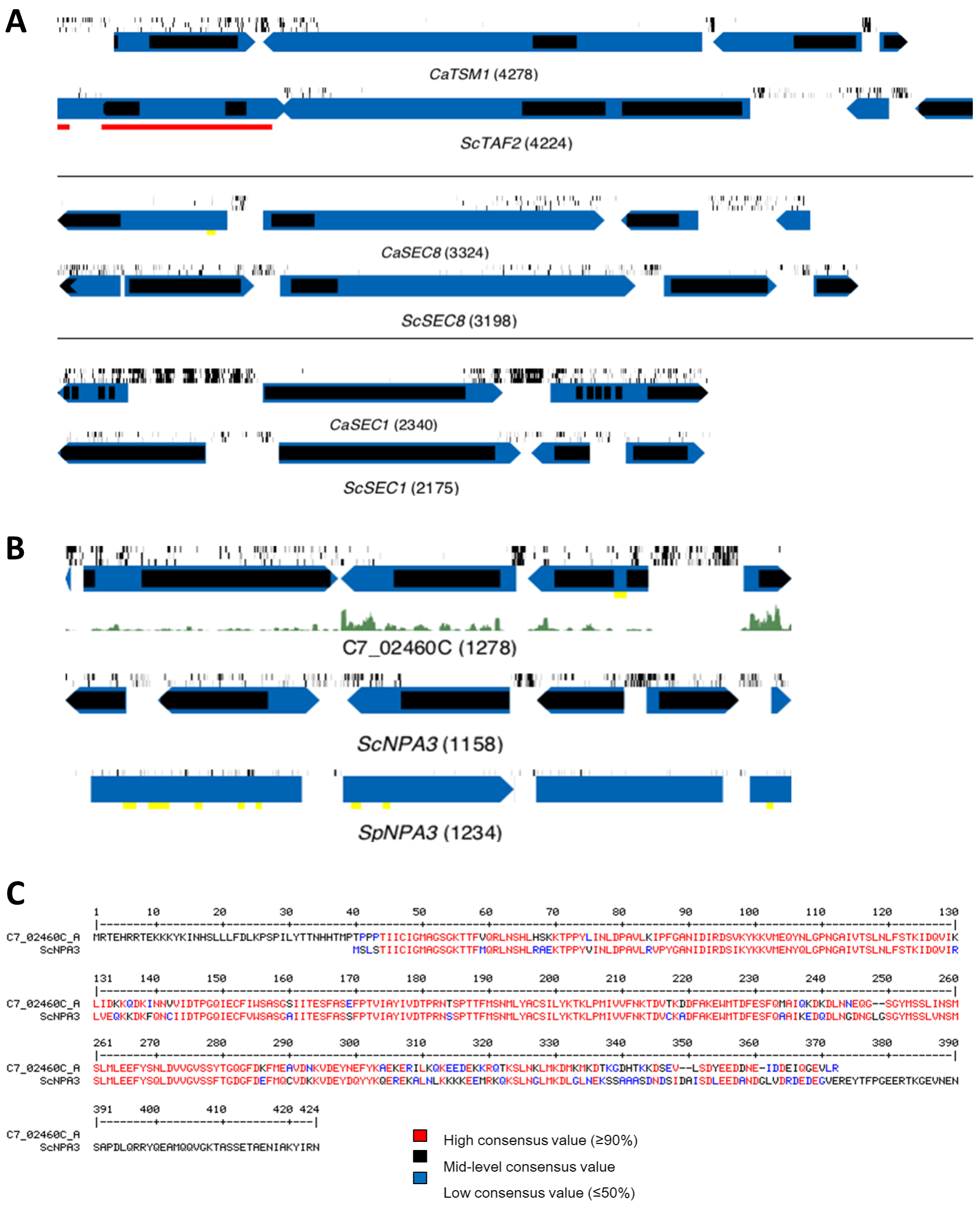

Supplement: FIG S2 [file mbo005184136sf2.tif]

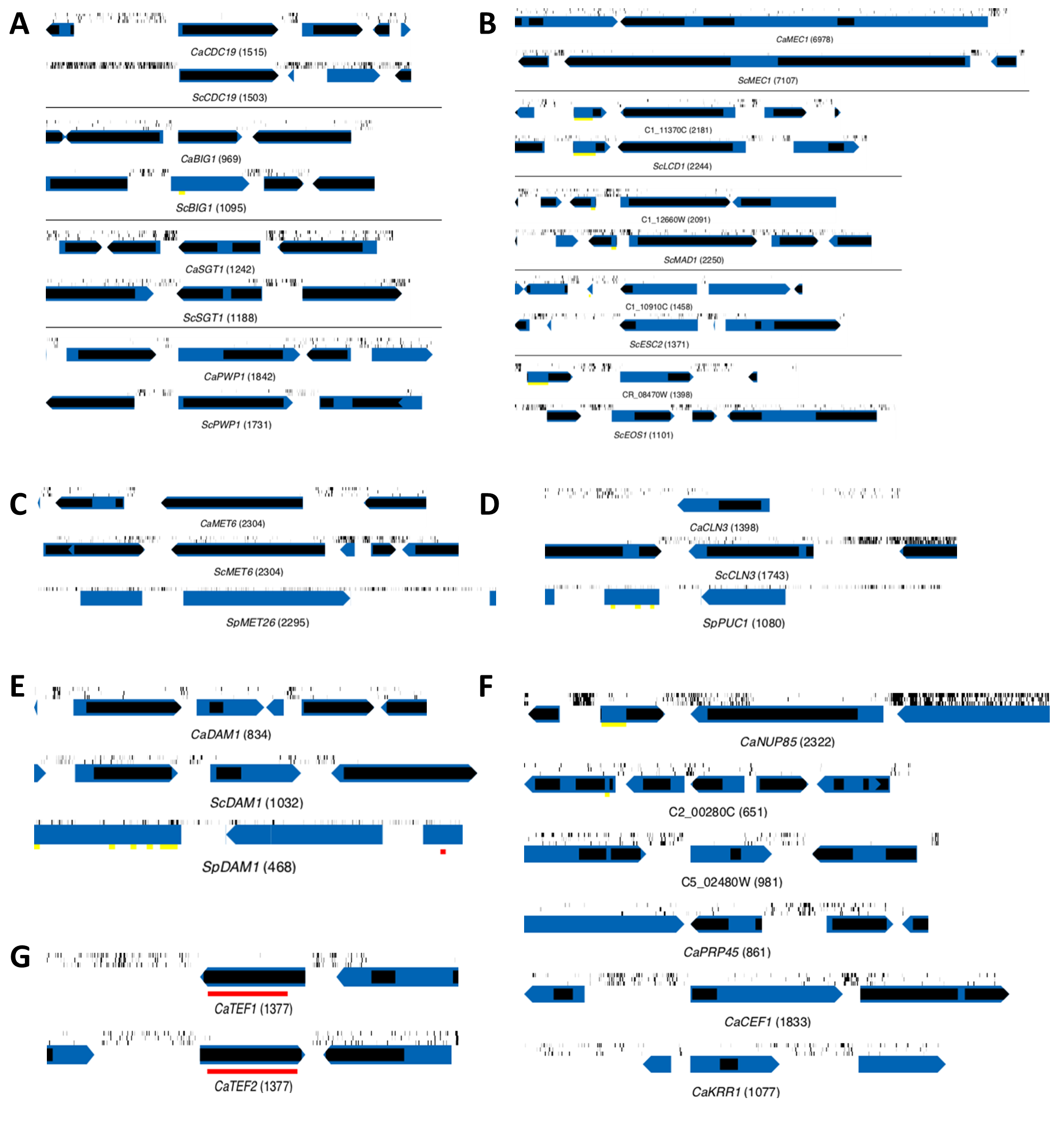

Supplement: FIG S4 [file mbo005184136sf4.tif]

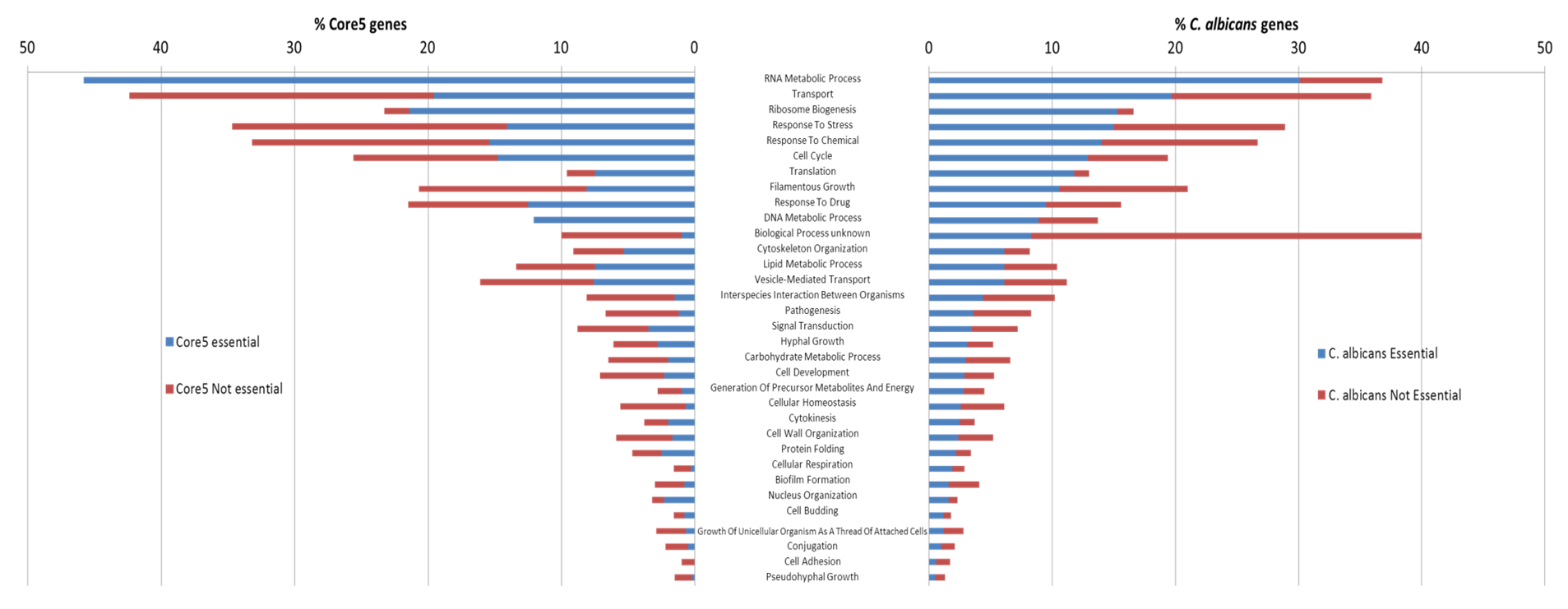

Supplement: FIG S3 [file mbo005184136sf3.tif]
