# Supplementary material for: Gene Essentiality Analyzed by In Vivo Transposon Mutagenesis and Machine Learning in a Stable Haploid Isolate of Candida albicans
Source: mBio. 2018 Oct 30;9(5):e02048-18. doi: 10.1128/mBio.02048-18 (PMC6212825; doi:10.1128/mBio.02048-18)
Supplement: TABLE S1 [file mbo005184136st1.pdf]

Table S1. Annotated features in *C. albicans*

blocked_reading_frame
blocked_reading_frame|transposable element gene
centromere
long_terminal_repeat
ncRNA|Uncharacterized
ncRNA|Verified
ORF|Alternatively spliced|Uncharacterized
ORF|Alternatively spliced|Verified
ORF|Dubious
ORF|Uncharacterized
ORF|Uncharacterized|transposable element gene
ORF|Verified
ORF|Verified|transposable element gene
pseudogene
pseudogene|Uncharacterized
repeat_region
retrotransposon
rRNA|Verified
snoRNA|Uncharacterized
snRNA|Uncharacterized
tRNA|Uncharacterized
tRNA|Verified
