# Supplementary material for: Gene Essentiality Analyzed by In Vivo Transposon Mutagenesis and Machine Learning in a Stable Haploid Isolate of Candida albicans
Source: mBio. 2018 Oct 30;9(5):e02048-18. doi: 10.1128/mBio.02048-18 (PMC6212825; doi:10.1128/mBio.02048-18)
Supplement: TABLE S2 [file mbo005184136st2.pdf]

Table S2. Number of insertions and reads in regions with low and high likelihood of nucleosome occupancy

Low and high nucleosome occupancy were derived from Tsankov et al. 2010.
All log read values were normalized to the chromosome length

Library	Chromosome	Hits in Low nucleosome occupancy region	Hits in high nucleosome occupancy region	Log Reads in Low nucleosome occupancy regions	Log reads in high nucleosome occupancy regions
3	Ca22chr1A_C_albicans_SC5314	0.100	0.068	0.056	0.040
3	Ca22chr2A_C_albicans_SC5314	0.102	0.068	0.057	0.039
3	Ca22chr3A_C_albicans_SC5314	0.231	0.183	0.141	0.116
3	Ca22chr4A_C_albicans_SC5314	0.124	0.085	0.070	0.050
3	Ca22chr5A_C_albicans_SC5314	0.132	0.096	0.075	0.057
3	Ca22chr6A_C_albicans_SC5314	0.116	0.084	0.066	0.050
3	Ca22chr7A_C_albicans_SC5314	0.159	0.119	0.093	0.071
3	Ca22chrRA_C_albicans_SC5314	0.087	0.061	0.049	0.036
7	Ca22chr1A_C_albicans_SC5314	0.067	0.045	0.032	0.022
7	Ca22chr2A_C_albicans_SC5314	0.067	0.046	0.032	0.022
7	Ca22chr3A_C_albicans_SC5314	0.160	0.127	0.083	0.066
7	Ca22chr4A_C_albicans_SC5314	0.081	0.056	0.038	0.027
7	Ca22chr5A_C_albicans_SC5314	0.088	0.063	0.043	0.031
7	Ca22chr6A_C_albicans_SC5314	0.077	0.057	0.037	0.028
7	Ca22chr7A_C_albicans_SC5314	0.105	0.078	0.052	0.037
7	Ca22chrRA_C_albicans_SC5314	0.058	0.040	0.027	0.019
11	Ca22chr1A_C_albicans_SC5314	0.070	0.046	0.038	0.026
11	Ca22chr2A_C_albicans_SC5314	0.070	0.047	0.038	0.027
11	Ca22chr3A_C_albicans_SC5314	0.173	0.135	0.101	0.082
11	Ca22chr4A_C_albicans_SC5314	0.087	0.059	0.047	0.034
11	Ca22chr5A_C_albicans_SC5314	0.093	0.066	0.051	0.038
11	Ca22chr6A_C_albicans_SC5314	0.081	0.059	0.045	0.034
11	Ca22chr7A_C_albicans_SC5314	0.116	0.083	0.065	0.047
11	Ca22chrRA_C_albicans_SC5314	0.059	0.041	0.032	0.023
