# Supplementary material for: Gene Essentiality Analyzed by In Vivo Transposon Mutagenesis and Machine Learning in a Stable Haploid Isolate of Candida albicans
Source: mBio. 2018 Oct 30;9(5):e02048-18. doi: 10.1128/mBio.02048-18 (PMC6212825; doi:10.1128/mBio.02048-18)
Supplement: TEXT S1 [file mbo005184136s1.docx]

**Supplementary figures**

**Figure S1** Virulence of haploid reads, transposon insertion read maps and machine learning performance. (A) Virulence of *C. albicans* haploid strains vs SC5314 wild type in a mouse systemic infection model: Five mice per *Candida* strain (SC5314 and haploids YJBT1792 and YJBT1082) were injected via tail vein with 2 × 10^5^ cells. The mice were monitored daily for two weeks post-inoculation for weight loss and signs of clinical illness. All the mice injected with haploid strains, with one exception, were healthy until the end of the study period. Mice showing more than 20% weight loss or a clinical illness score of 2.5 were culled. (B) Maps of transposon insertion reads (number of times each site was sequenced) for the whole genome and Chr7. Distribution of insertion sites (hits) for pooled library used in this study (#3, #7 and #11) as in Figure 2 (C) Performance of machine-learning Random Forest classifier with 5-fold cross validation tests. ROC curve for prediction of *C. albicans* gene set, AUC=0.997. FPR and TP rates are shown in Table 3 for all three organisms.

Supplementary note for Fig. S1A: virulence of the *Candida* strains was assessed using a low inoculum of 2 × 10^5^ cells/mouse because higher inocula (e.g., 10^6^ SC5314 cells per mouse) leads to rapid onset of illness and is less discriminating in assessing relative virulence of strains [16]. Mice injected with a wild type strain displayed quicker progression of disease compared to those injected with haploid strains. All the mice injected with haploid strains, with one exception, were healthy until the end of the study period, indicating that haploid *Candida* strains were attenuated in virulence compared to their parental wild type strain.

**Figure S2**. Hit maps of *C. albicans* and *S. cerevisiae* genes with conserved organization of essential domains, examples of mis-annotated *C. albicans* ORFs and alignment of proteins they predict. (A) Hit maps of *C. albicans* and *S. cerevisiae* genes with conserved organization of essential domains. Symbols are as in Fig. 2. Red bars indicate regions ignored because they include repeated sequences. (B) *CaTn* data reveals mis-annotation of several *C. albicans* ORFs. Alignment of hit maps for *C. albicans* gene C7_02460C and its orthologs *ScNPA3,* and *SpNPA3*. Some insertions in C7_02460C are near the extreme N-terminus of the ORF and the transcript in the RNAseq data (green histogram, [1]) initiates downstream of the initiation codon. Note that the annotated *C. albicans* protein initiates 40 amino acids before the initiation codon of the *S. cerevisiae* coding sequence. We suggest that the correct ORF starts at Chr7 coordinate 532,126. Symbols are as in main Fig. 2. Note that the *C. albicans* and *S. cerevisiae* genes appear to have a C-terminal domain that can tolerate insertions. (C) Alignment of predicted proteins encoded by C7_02460C (top line) and *ScNPA3* (bottom line); note that similarity begins at amino acid 44. Blue letters, identical amino acids with low-level consensus value; red letters, high consensus value (≥90%); black letters, mid-level consensus value.

**Figure S3.** Gene ontology (GO) term analysis of Core5 essential genes and Core5 non-essential genes. GO ‘biological process’ terms using GO slim analysis performed using (<http://www.candidagenome.org/cgi-bin/GO/goTermMapper>). X-axis, proportion of genes in the given ‘biological process’ ontology term, expressed as a percentile of total genes in that term.

Supplementary note: In general, the distribution of Gene Ontology (GO) terms amongst the *C. albicans* essential genes (*Ca*Ess) and the Core5 essential gene set was similar (Fig. S3). Notable differences include a larger proportion of *Ca*Tn-Ess genes with “no known biological process”, and with GO terms “pathogenesis”, “adhesion” and “biofilm formation”, relative to the Core5 genes, which reflects what is known about the biology of C. albicans, as well as what types of studies have been performed in C. albicans relative to the model yeasts.

**Figure S4.** Specific examples of *C. albicans* genes whose contradict those in S. cerevisiae and/or *S. pombe*.

(A) Examples of conditionally essential genes in *C. albicans* and their orthologs in *S. cerevisiae.*  Symbols are as in main Fig. 2.

Supplementary note: Secondary suppressor mutations are known to accumulate in some deletion mutants in *S. cerevisiae* [19] such as *VMA6, VPS52* and *VPH2* genes, which were among the genes that were *Sc*∆-NE yet *Sc*Tn-Ess. Because the Tn insertion mutants were analyzed early after mutations were generated (after two 24h passages) they apparently did not undergo sufficient selection to accumulate this type of suppressor mutation.

Conditional essentiality is another common reason for different outcomes in studies of essentiality. For example, outliers *CDC19* and *BIG1* are essential under the conditions used to select for transposon insertions (medium with glucose and without sorbitol, respectively) and thus were predicted essential in transposon studies (Fig. S4A). These two genes should tolerate transposon insertions if grown either in the absence of glucose (*cdc19)* or the presence of sorbitol (*big1)*. Genes in other biosynthetic pathways are also likely to be conditional depending on the components included in the medium. Genes in the adenine biosynthesis pathways (*ScADE1, ScADE3* and *ScADE6 CaADE1, CaADE6* and *CaADE17)* were predicted *Sc*Tn-Ess and *Ca*Tn-Ess, respectively, presumably because of the selection for adenine prototrophy in both screens for transposition events. Other genes may be conditional because nourseothricin was used to select for the *Ds-NAT1.* Similarly, *SpURA6* was predicted *Sp*Tn-Ess because it was used in the Hermes screen.

Domain-essential genes clearly contributed to the outlier classes as well, with 38% (29/77) of the *Sc*∆-Ess/ScTn-NE genes and 18% (21/117) of *Sc*∆-NE/*Sc*Tn-Ess genes being ‘domain-essential’ based on the 'Domain likelihood score' [11] (e.g., *NUP170,* Fig. 3A. Interestingly, among the 77 *Sc*∆-Ess/*Sc*Tn-NE outliers, were genes (*BCD1, TIF6*) that are not essential in the *S. cerevisiae* Sigma 1278b strain [6, 9]. Thus, essentiality of some genes is subject to strain-specificity issues as well.

In the *Sp*Tn data 50 genes were highlighted as *Sp*Tn-NE that were *Sp*∆-Ess [8], presumably because they grow very slowly. Of these, 40% were found to have a wild-type copy of the relevant gene, and thus were not *bona fide* deletion mutants [8]. 48 of these 50 genes were judged *Sp*Tn-NE by the ML classifier. Of these, 13 were clearly *Ca*Tn-NE. Five of them encode mitochondrial proteins, suggesting that the disruption of mitochondrial proteins may not cause immediate loss of viability, allowing these hits to be detected in transposon analysis while they might be lost during the more rigorous selection for viable spores from deletion mutants.

(B) Hit maps of cell cycle checkpoint genes with contradictory predictions in CaTn vs ScTn analyses. *MEC1* and *LCD1* (C1_11379C) are ScTn-Ess/CaTn-NE*; MAD1* (C1_12660W) and *ESC2* (C1_10910C) are ScTn-NE/CaTn-Ess; *EOS1* (CR_08470W) is essential in both ScTn and CaTn. Symbols are as in Fig. 2.

Supplementary note: Two cell cycle checkpoint genes, *CaMEC1* and C1_11370C (similar to *ScMEC1* and *ScDDC2/LCD1)* are essential in *S. cerevisiae* and not in *S. pombe* or *C. albicans*. In *S. cerevisiae*, the essentiality of *MEC1* and *DDC2* can be relieved by mutations that alter the dNTP concentration in the cell [5] and the essentiality of *MEC1* is strain-dependent [6, 9]. This suggests that there are differences in the relationship between dNTP levels and cell cycle progression in *C. albicans (*and *S. pombe*) relative to the classic *S. cerevisiae* model system. In *S. cerevisiae* Ddc2 brings Mec1 (the ATR) to sites of DNA damage. We suggest that either the constitutive dNTP concentration or ribonucleotide reductase activity is higher, and/or the degree to which dNTPs are required for checkpoint function are much lower than in *S. cerevisiae.*

Because *S. cerevisiae* and *C. albicans* divide by budding, while *S. pombe* divides via fission, some cell cycle processes are much more similar between the two budding yeasts. Yet, two cell cycle checkpoints, *MAD1* and *ESC2* (Fig. S4B)*,* were definitively non-essential in *S. cerevisiae* (*Sc*∆-NE/*Sc*Tn-NE) and yet were essential here (*Ca*Tn-Ess). Similar processes were identified for essentiality differences between *S. pombe and S. cerevisiae* [10]. In *S. cerevisiae*, *Sc*Mad1 protein forms a complex with *Sc*Mad2 and inhibits the anaphase promoting complex if the checkpoint is activated. Mad2 is not essential in either *S. cerevisiae* or *C. albicans*. *Sc*Esc2 has a role in the intra-S DNA damage checkpoint and mitotic sister chromatid cohesion. Interestingly, *EOS1* is *Sc*∆-NE, yet was predicted essential in both *Sc*Tn-Ess and *Ca*Tn-Ess transposon screens (with RF prediction scores of 0.98 and 1 respectively). In *S. cerevisiae*, *EOS1* is synthetic lethal with *MAD1*. The roles of *Ca*Mad1 and *Ca*Esc2 (C1_10910C_A) and *CaEOS1* (CR_08470W_A) have not been studied. However, *CaEOS1* transcripts have been differentially regulated in two biofilm models [13, 14]. We suggest that differences in essentiality reflect differences in the relative importance of several cell cycle complexes and the checkpoints that regulate them in the different yeasts.

(C) Hit maps of *MET6* (C) and *CLN3* (D) orthologs from all three yeasts. *C. albicans* genes are clearly essential while the model yeast orthologs are not, reflecting biological differences between *C. albicans* and model yeast biology. Symbols are as in Fig. 2.

Supplemental text for Fig. S4C&D: *MET6* encodes a cobalamin-independent methionine synthase that localizes to the nucleus in respiratory yeasts*,* yet is cytoplasmic in *S. cerevisiae* [17]. This supports the idea that *MET6* likely has a secondary role that renders it essential in *C. albicans* [18]. Interestingly, *MET6* is conserved in other pathogens and not in humans, making it a potential target for antifungal drugs (discussed below).

*(D) CaCLN3,* a regulator of cell cycle progression and filamentous growth in *C. albicans* is essential by all measures: *Ca*Tn, UAU1, repression [15] and classic disruption approaches [3, 4].*CLN3* repression caused increased pseudohyphal-like growth, indicative of cell cycle progression defects [7]. By contrast, *Sc*Cln3 is a non-essential cyclin-dependent kinase that initiates the G1 to S transition in response to cell size and nutrient status. In *S. pombe,* the *CLN3* ortholog, *Puc1,* is also redundant with other cyclins. Thus, unlike in *S. cerevisiae* and *S. pombe*, *CaCLN3* is critical for *C. albicans* viability, which provides an explanation for the more direct connections between the regulation of G1 entry and cell cycle progression in *C. albicans* [3], a process that does not occur in the model yeasts.

(E) Hit maps of *DAM1* orthologs in the three yeasts. *DAM1* was determined essential in *S. cerevisiae* and *C. albicans* by deletion studies, yet is predicted non-essential in TnSeq studies in both organisms. Symbols are as in Fig. 2; red box indicates region of repeated sequence that is not uniquely mapped by Illumina sequencing.

*C. albicans* and *S. cerevisiae* kinetochores and centromeres have fundamental differences [2]: *S. cerevisiae* have sequence-dependent point centromeres that attach to a single kinetochore microtubule via the DAM1/DASH complex. By contrast, *C. albicans* has small regional centromeres with epigenetic inheritance, can have more than one microtubule per kinetochore [2, 20] and the DAM1/DASH complex is less critical if the number of microtubules are increased via overexpression of the centromeric histone [2]. Other genes predicted to be components of the DASH complex (*ASK1, SPC19, DAM1/DAD1, DAM2, DAD3, DAD4, SPC19)* are essential in both *C. albicans* and *S. cerevisiae* [2, 20] (Dataset 2D and Fig. S4E;[12]; <https://www.yeastgenome.org/>). Interestingly, in *S. pombe, dam1, dad1, spc34, ask1, dam2, duo1* and *spc19* were also non-essential, although some were conditionally essential at slightly higher temperature [10].

(F) Hit maps of genes essential in both model yeasts, yet predicted non-essential in *C. albicans*. In this case, an apparent hit-free domains that may have been missed by the classifier.

(G) Example of two genes that were excluded from the analysis due to high sequence similarity.

(H) Example of short gene and its insertion profile in a region of the genome that had low insertion frequencies. Symbols are as in Fig. S2; red indicates repeated regions that could not be mapped accurately.

1. Bruno, V.M., et al.,2010. *Comprehensive annotation of the transcriptome of the human fungal pathogen Candida albicans using RNA-seq.* Genome Res, **20**(10): p. 1451-8. DOI: 10.1101/gr.109553.110.

2. Burrack, L.S., S.E. Applen, and J. Berman,2011. *The requirement for the Dam1 complex is dependent upon the number of kinetochore proteins and microtubules.* Curr Biol, **21**(10): p. 889-96. DOI: 10.1016/j.cub.2011.04.002.

3. Chapa y Lazo, B., S. Bates, and P. Sudbery,2005. *The G1 cyclin Cln3 regulates morphogenesis in Candida albicans.* Eukaryot Cell, **4**(1): p. 90-4. DOI: 10.1128/EC.4.1.90-94.2005.

4. Davis, D.A., et al.,2002. *Candida albicans Mds3p, a conserved regulator of pH responses and virulence identified through insertional mutagenesis.* Genetics, **162**(4): p. 1573-81.

5. Desany, B.A., et al.,1998. *Recovery from DNA replicational stress is the essential function of the S-phase checkpoint pathway.* Genes Dev, **12**(18): p. 2956-70.

6. Dowell, R.D., et al.,2010. *Genotype to Phenotype: A Complex Problem.* Science, **328**(5977): p. 469-469. DOI: 10.1126/science.1189015.

7. Finley, K.R., et al.,2008. *Dynein-dependent nuclear dynamics affect morphogenesis in Candida albicans by means of the Bub2p spindle checkpoint.* J Cell Sci, **121**(Pt 4): p. 466-76. DOI: 10.1242/jcs.015172.

8. Guo, Y., et al.,2013. *Integration profiling of gene function with dense maps of transposon integration.* Genetics, **195**(2): p. 599-609. DOI: 10.1534/genetics.113.152744.

9. Hou, J., et al.,2018. *Genetic Network Complexity Shapes Background-Dependent Phenotypic Expression.* Trends Genet, **34**(8): p. 578-586. DOI: 10.1016/j.tig.2018.05.006.

10. Kim, D.U., et al.,2010. *Analysis of a genome-wide set of gene deletions in the fission yeast Schizosaccharomyces pombe.* Nat Biotechnol, **28**(6): p. 617-623. DOI: 10.1038/nbt.1628.

11. Michel, A.H., et al.,2017. *Functional mapping of yeast genomes by saturated transposition.* Elife, **6**. DOI: 10.7554/eLife.23570.

12. Mielich, K., et al.,2018. *Maize Transposable Elements Ac/Ds as Insertion Mutagenesis Tools in Candida albicans.* G3 (Bethesda), **8**(4): p. 1139-1145. DOI: 10.1534/g3.117.300388.

13. Nett, J.E., et al.,2009. *Time Course Global Gene Expression Analysis of an In Vivo Candida Biofilm.* The Journal of Infectious Diseases, **200**(2): p. 307-313. DOI: 10.1086/599838.

14. Nobile, C.J., et al.,2012. *A recently evolved transcriptional network controls biofilm development in Candida albicans.* Cell, **148**(1-2): p. 126-38. DOI: 10.1016/j.cell.2011.10.048.

15. O'Meara, T.R., et al.,2015. *Global analysis of fungal morphology exposes mechanisms of host cell escape.* Nat Commun, **6**: p. 6741. DOI: 10.1038/ncomms7741.

16. Odds, F.C., L. Van Nuffel, and N.A. Gow,2000. *Survival in experimental Candida albicans infections depends on inoculum growth conditions as well as animal host.* Microbiology, **146 ( Pt 8)**: p. 1881-9. DOI: 10.1099/00221287-146-8-1881.

17. Sahu, U., et al.,2017. *Methionine synthase is localized to the nucleus in Pichia pastoris and Candida albicans and to the cytoplasm in Saccharomyces cerevisiae.* J Biol Chem, **292**(36): p. 14730-14746. DOI: 10.1074/jbc.M117.783019.

18. Suliman, H.S., D.R. Appling, and J.D. Robertus,2007. *The gene for cobalamin-independent methionine synthase is essential in Candida albicans: a potential antifungal target.* Arch Biochem Biophys, **467**(2): p. 218-26. DOI: 10.1016/j.abb.2007.09.003.

19. Teng, X., et al.,2013. *Genome-wide consequences of deleting any single gene.* Mol Cell, **52**(4): p. 485-94. DOI: 10.1016/j.molcel.2013.09.026.

20. Thakur, J. and K. Sanyal,2011. *The essentiality of the fungus-specific Dam1 complex is correlated with a one-kinetochore-one-microtubule interaction present throughout the cell cycle, independent of the nature of a centromere.* Eukaryot Cell, **10**(10): p. 1295-305. DOI: 10.1128/EC.05093-11.
