# Supplementary material for: Gene Essentiality Analyzed by In Vivo Transposon Mutagenesis and Machine Learning in a Stable Haploid Isolate of Candida albicans
Source: mBio. 2018 Oct 30;9(5):e02048-18. doi: 10.1128/mBio.02048-18 (PMC6212825; doi:10.1128/mBio.02048-18)
Supplement: TABLE S3 [file mbo005184136st3.pdf]

Table S3. Riboflavin biosynthesis pathway analysis

(1) O'Meara et al. 2015 Nature Comm.

Ca standard name	Common name	*Ca* Tn-Ess	Tet Repression study (1)	ESC-Ess	ScΔ-Ess	ScTn-Ess	SpΔ-Ess	SpTn-Ess
CR_02990C_A	RIB1	Ess	Ess	Ess	NE	Ess	Ess	Ess
C1_12360C_A	RIB3	Ess	NE	NE	Ess	Ess	Ess	Ess
C1_05560W_A	RIB4	Ess	NE	NE-repr	NE	Ess	Ess	Ess
C5_05300W_A	RIB5	NE	NE	NE	Ess	Ess	Ess	Ess
C1_12810W_A	RIB7	Ess	NE	NE	Ess	Ess	Ess	NE
CR_03740C_A	FMN1	Ess	NE	NE	Ess	Ess	Ess	Ess
C1_08650C_A	FAD1	Ess	no data	Ess	Ess	Ess	NE	Ess
