# Supplementary material for: Gene Essentiality Analyzed by In Vivo Transposon Mutagenesis and Machine Learning in a Stable Haploid Isolate of Candida albicans
Source: mBio. 2018 Oct 30;9(5):e02048-18. doi: 10.1128/mBio.02048-18 (PMC6212825; doi:10.1128/mBio.02048-18)
Supplement: TABLE S4 [file mbo005184136st4.pdf]

Table S4A. Yeast strains

Strain number	Ploidy	Description	Source
YJB-T 1 (SC5314)	Diploid	*C. albicans* type strain (Gillum et al. 1984)	Lab stocks
YJB-T 900 (GZY896)	Haploid	*MTL **a** ura3Δ::imm434 his4 gal1Δ::ura3Δ::HIS4*	(Mielich et al. 2018)
YJB-T 1792	Haploid	*MTL **a** ura3Δ::imm434 his4 gal1Δ::ura3Δ::HIS4 NEUT5L region ::Ac URA3*	This study
YJB-T 1795	Haploid	*MTL **a** ura3Δ::imm434 his4 gal1Δ::ura3Δ::HIS4 ade2::Ds NAT1*	This study
YJB-T 1081	Haploid	MTL***a*** *ura3Δ::imm434 his4 gal1Δ::ura3Δ::HIS4 ade2::Ds NAT1 NEUT5L region ::Ac URA3*	This study
YJB-T 1082	Haploid	MTL***a*** *ura3Δ::imm434 his4 gal1Δ::ura3Δ::HIS4 ade2::Ds NAT1 NEUT5L region ::Ac URA3*	This study
YJB-T 257 (GZY803)	Haploid	MTLα his4 ura3Δ::HIS4	(Hickman et al. 2013)
YJB-T 2743	Haploid	MTLα his4 ura3Δ::HIS4 ade2::Ds NAT1 NEUT5L region ::Ac URA3	This study
YJB 12801	Haploid	Haploid I *MTL **a** , his4, galΔ, ade2*	(Hickman et al. 2013)
YJB 12881	Haploid	Haploid XI *MTL **a** , his4, galΔ*	(Hickman et al. 2013)

Table S4B. Plasmids

Plasmid name	Description	Source
BJB-T 133 pRK402	Ds plasmid with *NAT1* marker	This study
BJB-T 135 pKM300	Ac plasmid with *URA3* marker	(Mielich et al. 2018)

Table S4C. Primers

Primer No.	Sequence	Purpose
BP117	GTTTTTGGAGTATTGTGATGGTAT	Check Ds integration/excision
BP118	TTTCGTTGTTGTTTCTTATTCTGGT	Check Ds integration
BP119	TCATCATCTGGGAAAACCTTAGTC	Check Ds integration
BP120	CCAAACTTTTCCCCAATGTGTAAC	Check Ds integration/excision
BP104	GGTGATGGATTAGGACAACA	Check Ac integration
BP161	AAAAGGCCTGATAAGGAGAGATCCATTAAGAGCA	Check Ac integration
BP664	AATGATACGGCGACCACCGAGATCTACACTCTTTCCCTAC ACGACGCTCTTCCGATCTNNNNNNGTATTTTACCGACCG	F_Tn specific primer with P5 and RD1 Illumina sequence
BP724	Phos/GATCGGAAGAGCACACGTCTGAACTCCAGTCA	Adapter sequence for Illumina
BP725	ACGCTCTTCCGATC*T	Adapter sequence for Illumina
BP847	CAAGCAGAAGACGGCATACGAGAT<u>CGTGAT</u>GTGACTGG AGTTCAGACGTGTGCTCTTCCGATCT	R_Tnseq_index1 with P7 and RD2 Illumina sequence

BP770	CAAGCAGAAGACGGCATACGAGATACATCGGTGACTGGAGTTCAGACGTGTGCTCTTCCGATCT	R_Tnseq_index2 with P7 and RD2 Illumina sequence
BP848	CAAGCAGAAGACGGCATACGAGATGCCTAAGTGACTGGAGTTCAGACGTGTGCTCTTCCGATCT	R_Tnseq_index3 with P7 and RD2 Illumina sequence
BP771	CAAGCAGAAGACGGCATACGAGATTGGTCAGTGACTGGAGTTCAGACGTGTGCTCTTCCGATCT	R_Tnseq_index4 with P7 and RD2 Illumina sequence
BP665	CAAGCAGAAGACGGCATACGAGATCACTGTGTGACTGGAGTTCAGACGTGTGCTCTTCCGATCT	R_Tnseq_index5 with P7 and RD2 Illumina sequence
BP666	CAAGCAGAAGACGGCATACGAGATATTGGCGTGACTGGAGTTCAGACGTGTGCTCTTCCGATCT	R_Tnseq_index6 with P7 and RD2 Illumina sequence
BP772	CAAGCAGAAGACGGCATACGAGATGATCTGGTGACTGGAGTTCAGACGTGTGCTCTTCCGATCT	R_Tnseq_index7 with P7 and RD2 Illumina sequence
BP849	CAAGCAGAAGACGGCATACGAGATTCAAGTGTGACTGGAGTTCAGACGTGTGCTCTTCCGATCT	R_Tnseq_index8 with P7 and RD2 Illumina sequence
BP850	CAAGCAGAAGACGGCATACGAGATTCAAGTGTGACTGGAGTTCAGACGTGTGCTCTTCCGATCT	R_Tnseq_index9 with P7 and RD2 Illumina sequence
BP851	CAAGCAGAAGACGGCATACGAGATAAGCTAGTGACTGGAGTTCAGACGTGTGCTCTTCCGATCT	R_Tnseq_index10 with P7 and RD2 Illumina sequence
BP852	CAAGCAGAAGACGGCATACGAGATGTAGCCGTGACTGGAGTTCAGACGTGTGCTCTTCCGATCT	R_Tnseq_index11 with P7 and RD2 Illumina sequence
BP773	CAAGCAGAAGACGGCATACGAGATTACAAGGTGACTGGAGTTCAGACGTGTGCTCTTCCGATCT	R_Tnseq_index12 with P7 and RD2 Illumina sequence
BP853	CAAGCAGAAGACGGCATACGAGATTTGACTGTGACTGGAGTTCAGACGTGTGCTCTTCCGATCT	R_Tnseq_index13 with P7 and RD2 Illumina sequence
BP854	CAAGCAGAAGACGGCATACGAGATGGAACTGTGACTGGAGTTCAGACGTGTGCTCTTCCGATCT	R_Tnseq_index14 with P7 and RD2 Illumina sequence
BP855	CAAGCAGAAGACGGCATACGAGATTGACATGTGACTGGAGTTCAGACGTGTGCTCTTCCGATCT	R_Tnseq_index15 with P7 and RD2 Illumina sequence
BP856	CAAGCAGAAGACGGCATACGAGATGGACGGGTGACTGGAGTTCAGACGTGTGCTCTTCCGATCT	R_Tnseq_index16 with P7 and RD2 Illumina sequence
BP857	CAAGCAGAAGACGGCATACGAGATGCGGACGTGACTGGAGTTCAGACGTGTGCTCTTCCGATCT	R_Tnseq_index18 with P7 and RD2 Illumina sequence
BP858	CAAGCAGAAGACGGCATACGAGATTTTCACGTGACTGGAGTTCAGACGTGTGCTCTTCCGATCT	R_Tnseq_index19 with P7 and RD2 Illumina sequence
BP859	CAAGCAGAAGACGGCATACGAGATGGCCACGTGACTGG	R_Tnseq_index20 with P7 and RD2

BP859	AGTTCAGACGTGTGCTCTTCCGATCT	Illumina sequence
BP860	CAAGCAGAAGACGGCATACGAGATCGAAACGTGACTGG<br>AGTTCAGACGTGTGCTCTTCCGATCT	R_Tnseq_index21 with P7 and RD2<br>Illumina sequence
BP861	CAAGCAGAAGACGGCATACGAGATCGTACGGTGACTGG<br>AGTTCAGACGTGTGCTCTTCCGATCT	R_Tnseq_index22 with P7 and RD2<br>Illumina sequence
BP862	CAAGCAGAAGACGGCATACGAGATCCACTCGTGACTGGA<br>GTTCAGACGTGTGCTCTTCCGATCT	R_Tnseq_index23 with P7 and RD2<br>Illumina sequence
BP863	CAAGCAGAAGACGGCATACGAGATATCAGTGTGACTGG<br>AGTTCAGACGTGTGCTCTTCCGATCT	R_Tnseq_index25 with P7 and RD2<br>Illumina sequence
BP864	CAAGCAGAAGACGGCATACGAGATAGGAATGTGACTGG<br>AGTTCAGACGTGTGCTCTTCCGATCT	R_Tnseq_index27 with P7 and RD2<br>Illumina sequence
